# Morbidity, Prognostic Factors, and Competing Risk Nomogram for Combined Hepatocellular-Cholangiocarcinoma

**DOI:** 10.1155/2021/3002480

**Published:** 2021-12-10

**Authors:** Xiaoyuan Chen, Yiwei Lu, Xiaoli Shi, Xuejiao Chen, Dawei Rong, Guoyong Han, Long Zhang, Chuangye Ni, Jie Zhao, Yun Gao, Xuehao Wang

**Affiliations:** ^1^School of Medicine, Southeast University, Nanjing, China; ^2^Hepatobiliary Center, The First Affiliated Hospital of Nanjing Medical University, Nanjing, China; ^3^Key Laboratory of Liver Transplantation, Chinese Academy of Medical Sciences, Nanjing, China; ^4^NHC Key Laboratory of Living Donor Liver Transplantation (Nanjing Medical University), Nanjing, China; ^5^Department of General Surgery, The Affiliated Jiangning Hospital of Nanjing Medical University, Nanjing, China

## Abstract

**Background:**

Combined hepatocellular-cholangiocarcinoma (CHC) is a rare and heterogeneous histological subtype of primary liver cancer, which is still poorly understood. This study aimed to describe the epidemiological and clinical features, investigate the prognostic indicators, and develop a competing risk nomogram for CHC.

**Methods:**

The study cohort was taken from the Surveillance, Epidemiology, and End Results database. The annual percent change (APC) in incidence was calculated using the joinpoint regression. The nomogram was developed based on multivariate competing risk survival analyses and validated by calibration curves. Akaike information criterion, Bayesian information criterion, Harrell's C-index, and area under the receiver operating characteristic curves were obtained to compare prognostic performance. Decision curve analysis was introduced to examine the clinical value of the models.

**Results:**

The overall incidence of CHC was 0.062 per 100,000 individuals in 2004 and 0.081 per 100,000 individuals in 2018, with an APC of 1.0% (*P* > 0.05). CHC displayed intermediate clinicopathological features of hepatocellular carcinoma and intrahepatic cholangiocarcinoma. Race, tumor size, vascular invasion, extrahepatic invasion, distant metastasis, grade, surgery, and Metavir stage were confirmed as the independent predictors of cancer-specific survival. The constructed nomogram was well calibrated, which showed better discrimination power and higher net benefits than the current American Joint Committee on Cancer staging system. Patients with liver transplantation had better survival than those with hepatectomy, especially patients within the Milan Criteria (*P*=0.022 and *P*=0.015). There was no survival difference between liver transplantation and hepatectomy in patients beyond the Milan Criteria (*P*=0.340).

**Conclusion:**

The morbidity of CHC remained stable between 2004 and 2018. The constructed nomogram could predict the prognosis with good performance, which was meaningful to individual treatment strategies optimization. CHC patients should also be considered as potential liver transplantation recipients, especially those within the Milan Criteria, but the finding still needs more evidence to be further confirmed.

## 1. Introduction

Combined hepatocellular-cholangiocarcinoma (CHC), defined as primary liver carcinomas with both hepatocellular and cholangiocellular differentiation within the same tumor in the WHO classification of tumors of the digestive system (5^th^ edition), is a rare histological subtype of primary liver cancer (0.4%–14.2%). CHC combines clinicopathological and radiological characteristics from both hepatocellular carcinoma (HCC) and intrahepatic cholangiocarcinoma (ICC) [1–9]. Although several decades have passed since CHC was first reported by Allen and Lisa in 1949 [10], this cancer is still poorly understood due to its rarity and complexity. Therefore, the first aim of this study was to explore the epidemiology and clinical features of CHC initially.

The American Joint Committee on Cancer (AJCC) classifies CHC and ICC under the same category in the 8^th^ edition staging system. However, some studies have pointed out the sufficient differences in clinical features and outcomes between CHC and ICC, indicating that CHC should be considered as a separate entity for a unique staging system [11–14]. During the follow-up period, various events, so-called competing risks, may either hinder the observation or modify the occurrence chance of events of interest, including accidents and comorbidities. In this context, conventional survival analyses such as the Kaplan–Meier method and the standard Cox regression model were inappropriate [15]. Hence, the second objective of this study was to conduct a competing risk survival analysis and then develop a nomogram to predict the prognosis of CHC patients.

Compared with HCC and ICC, treatment of CHC is not yet standardized, and many therapeutic options have been proposed. Overall, surgery remains the cornerstone of therapy to potentially cure localized CHC [3, 5–8]. Traditionally, due to the ICC component and a high recurrence rate (38%–100%), CHC is recognized as a contraindication for liver transplantation (LT). Only a few CHC patients were treated with LT due to preoperative misdiagnosis of HCC [16–19]. In recent years, some researchers have focused on the therapeutic value of LT in CHC patients and drawn encouraging results, but the sample sizes were limited in most studies [20–25]. Consequently, another objective of this study was to investigate the role of LT in CHC patients utilizing a large-scaled population-based database.

## 2. Patients and Methods

### 2.1. Patients

This study is a retrospective cohort study. The data of patients diagnosed with CHC (ICD-O-3 Histology Code: 8180/3) from 2004 to 2018 were extracted from the Surveillance, Epidemiology, and End Results (SEER) research database (18 registries), which is an authoritative source of information on cancer incidence and survival in the United States and covers approximately 34.6% of the population. Data were downloaded with SEER∗Stat software (version 8.3.9; the SEER Program, https://seer.cancer.gov). The inclusion criteria were shown as follows: (1) age ≥ 18 years; (2) being diagnosed with CHC based on positive histology; (3) having evidence of primary tumor; (4) known cause of death and survival time. The stepwise extraction process from the SEER database is shown in Supplementary Figure S1.

This study followed the Declaration of Helsinki (as revised in 2013). The SEER database is a public database without personal identifying information. In this context, the ethical review was exempted, and no consent was needed in this study.

### 2.2. Definitions

Annual percentage change (APC) was utilized to describe age-adjusted trends in the incidence of CHC. Demographic and clinical factors of study patients were obtained from the SEER database. Characteristics for each patient included the year of diagnosis, age, sex, race, residence, income, alpha-fetoprotein (AFP), cancer history, tumor number, tumor size, surgery, AJCC staging system, grade, and Metavir stage. Cancer-specific survival (CSS) and cumulative incidence of cancer-specific death (CSD) were set as the primary outcomes. Because the 8^th^ edition of AJCC staging system was not applicable before 2018, each patient was restaged according to the fields of “CS Extension”, “CS Lymph Nodes,” and “CS Mets at DX” in the SEER database.

### 2.3. Statistics

The Joinpoint Regression Program (version 4.7.0; IMS; Calverton, MD, USA) was used to analyze the APC in CHC incidence from 2004 to 2018. With the hypothesis that the incidence changed at a constant percentage from the previous year, the curve was fitted using the joinpoint regression. The APC in each segment can be calculated [26]. Survival analyses were performed by univariate and multivariate competing risk models. The cumulative incidences of CSD and other cause-specific death (OCSD) were estimated using the cumulative incidence function (CIF) curves. Propensity score matching (PSM) was used to reduce selection bias between groups. A one-to-one match was performed by the nearest-neighbor method within 0.20 standard deviations between the two groups. Categorical variables were shown as numbers and compared using the chi-square test or Fisher's exact test.

The study cohort was randomly divided into the training and validation set with the ratio of 7 : 3 for external validation. A nomogram was constructed based on multivariate competing risk survival analyses to provide a visual tool for clinical use. Akaike information criterion (AIC), Bayesian information criterion (BIC), Harrell's C-index, and area under receiver operating curves (AUROC) were calculated to compare prognostic performances of the nomogram and AJCC staging system. Calibration curves to evaluate the predictive accuracy of models were plotted via bootstrapping with 1000 resamples. Decision curve analysis (DCA) to estimate the clinical utility of models was performed by quantifying the net benefits at different threshold probabilities [27]. A result was considered statistically significant when two-tailed *P* < 0.05. All statistical analyses were completed using *R* software (version 3.6.3; The *R* Foundation for Statistical Computing, http://www.r-project.org).

## 3. Results

### 3.1. Incidence Trends of Combined Hepatocellular-Cholangiocarcinoma

**Figure 1:**
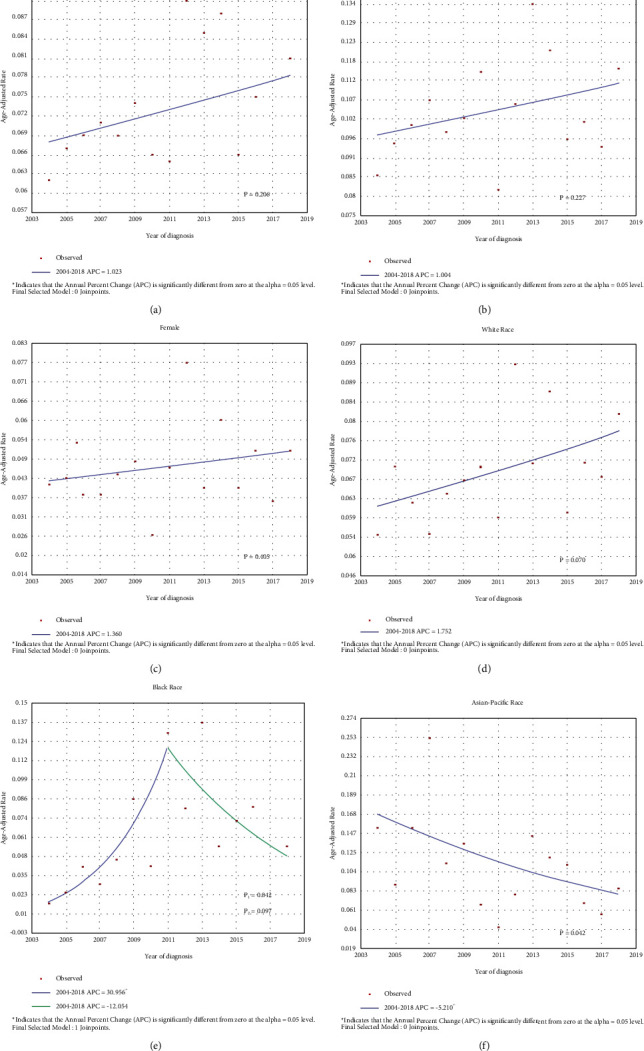
The morbidity trends of CHC by (a) overview, (b) male, (c) female, (d) White race, (e) Black race, and (f) Asian-Pacific race. CHC combined hepatocellular-cholangiocarcinoma; APC annual percentage change.

The overall incidence of CHC remained stable, and it was 0.062 per 100,000 individuals in 2004 and 0.081 per 100,000 individuals in 2018, with an APC of 1.0% [95% confidence interval (CI) = −0.6–2.7, *P* > 0.05, Figure 1(a)]. Then, the study population was divided into two subgroups according to sex. The incidence of CHC in males was 0.086 per 100,000 individuals in 2004 and 0.116 per 100,000 individuals in 2018. The APC was 1.0% (95% CI = −0.7–2.7, *P* > 0.05, Figure 1(b)) during the period. As for females, the incidence was 0.041 per 100,000 individuals in 2004 and 0.051 per 100,000 individuals in 2018, and the APC was 1.4% (95% CI = −2.0–4.9, *P* > 0.05, Figure 1(c)). Although the APC values were similar, there may be gender differences in the absolute incidence of CHC.

Data were further examined according to different races. In the Caucasian ethnicity, the trends of incidence were almost the same as the overall cohort (APC = 1.8%, 95% CI = −0.2–3.7, *P* > 0.05, Figure 1(d)). The incidence in the black race between 2004 and 2011 remained increasing with an APC of 31.0% (95% CI = 1.2–69.5, *P* < 0.05), and there was a sudden decline from 2012 to 2018 with an APC of -12.1% (95% CI = −24.8–2.8, *P* > 0.05, Figure 1(e)). In Asia-Pacific populations, the incidence of CHC has declined gradually (APC = −5.2%, 95% CI = −9.9∼−0.2, *P* < 0.05, Figure 1(f)), but the incidence was still 0.086 per 100,000 individuals in 2018.

### 3.2. Baseline Characteristics of Combined Hepatocellular-Cholangiocarcinoma

The baseline characteristics are shown in Table 1. A total of 736 patients were enrolled in this study, comprising 241 (32.7%) females and 495 males (67.3%). The median age at diagnosis was 63 [interquartile range (IQR): 57–71] years. Less than half (40.4%) of the patients underwent surgery, in which hepatectomy (61.8%) and LT (26.7%) were the first two treatment choices. Lymph node metastasis (LNM) was identified in 85 (11.5%) patients. About a quarter (25.5%) of patients were reported to have distant metastases. According to the AJCC staging system, the majority (64.3%) of patients were classified as pT1-pT2. Most of the patients with known clinical data had poorly differentiated or undifferentiated tumors (63.2%, 273/432) and liver cirrhosis (62.5%, 75/120).

The final follow-up was performed in November 2020, and 555 (75.4%) patients died during the follow-up period. The mean survival time was 20.2 ± 31.9 (IQR: 1–25) months. The 1 yr, 3 yr, and 5 yr cumulative incidences of CSD were 50.0%, 66.0%, and 71.4%, respectively. The 1 yr, 3 yr, and 5 yr cumulative incidences of OCSD were 6.8%, 8.9%, and 10.7%, respectively.

### 3.3. Development and Validation of the Nomogram

Further survival analyses were performed in 524 patients who had assessable primary tumors and definite surgery data. The study patients were randomly divided into the training set (*n* = 367) and the validation set (*n* = 157) with a ratio of 7 : 3. The baseline characteristics data of the training and validation set were displayed in Supplementary Table S1. As shown in Table 2, Table S2, and Supplementary Figure S2, the CIF curves showed that cancer history, tumor size, surgery, extrahepatic invasion, LNM, distant metastasis, and grade were found to be significantly associated with CSD (all *P* < 0.05). According to the multivariate competing risk analyses, race, tumor size, surgery, vascular invasion, extrahepatic invasion, distant metastasis, grade, and Metavir stage were confirmed as the independent prognostic indicators of CSD in the training set (all *P* < 0.05).

**Figure 2:**
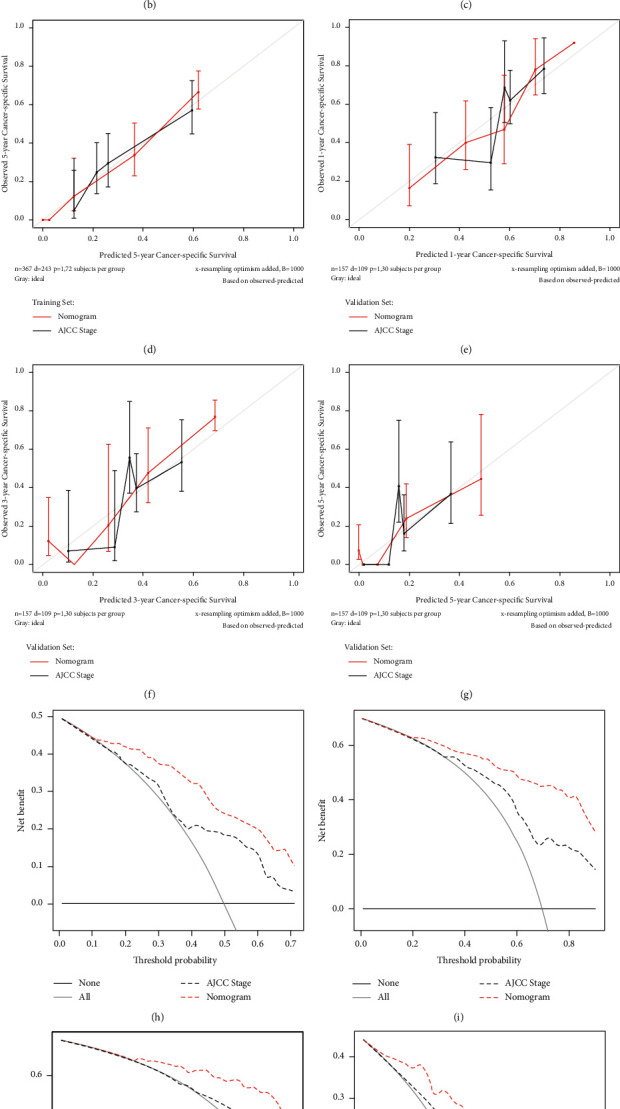
Development, validation, and comparison of the nomogram: (a) the nomogram to predict cancer-specific survival (CSS) developed from the training set based on competing risk analyses; (b–d) calibration curve analysis of nomogram and the current AJCC staging system (8^th^ edition) in the prediction of prognosis at 1-, 3-, 5-year point for CSS in the training set; (e–g) calibration curve analysis of nomogram and the current AJCC staging system (8^th^ edition) in the prediction of prognosis at 1-, 3-, 5-year point for CSS in the validation set; (h–j) decision curve analysis (DCA) of nomogram and the current AJCC staging system (8^th^ edition) in the prediction of prognosis at 1-, 3-, 5-year point for CSS in the training set; (k–m) DCA of nomogram and the current AJCC staging system (8^th^ edition) in the prediction of prognosis at 1-, 3-, 5-year point for CSS in the validation set. AJCC American Joint Committee on Cancer; LD local destruction; Hx hepatectomy; LT liver transplantation.

The nomogram was developed based on the independent prognostic indicators to predict cancer-specific survival of CHC patients (Figure 2(a)). The Harrell's C-indexes of the nomogram were 0.790 (95% CI = 0.761–0.820) in the training set and 0.736 (95% CI = 0.683–0.788) in the validation set, respectively. The calibration curves showed good consistency between the predicted and the observed CSS in both the training and validation set (Figures 2(b)–2(g)). The AUROC values were also performed as criteria to identify the reliability of the nomogram. As shown in Supplementary Figure S3, the 1 yr, 3 yr, and 5 yr of AUROC values of the nomogram were 0.857, 0.880, and 0.901 in the training set and 0.818, 0.882, and 0.730 in the validation set. The AIC and BIC values of the nomogram were also obviously lower than those of the AJCC staging system in both the training and validation set. Compared with the AJCC staging system, the nomogram showed a better discriminative capacity (*P* < 0.001, Table 3). To further estimate the clinical utility of models, DCAs were displayed in Figures 2(h)–2(m). The nomogram provided a better net benefit than “treat-all” or “treat-non” schemes and the AJCC staging system.

To further simplify the application of the nomogram, an online tool has been produced and published, which can be accessed through the following URL: https://chenxiaoyuan.shinyapps.io/CHC-DynNom/.

### 3.4. The Role of Liver Transplantation in Patients with Combined Hepatocellular-Cholangiocarcinoma

Surgery has been considered as an independent prognostic factor of CHC patients. However, as the second common surgical approach, the benefit of LT in CHC is still controversial. In this cohort, there were 79 (10.7%) CHC patients who underwent LT, in which 55 (69.6%) patients were within the Milan Criteria, 9 (11.4%) patients were beyond the Milan Criteria, and 15 patients had unknown data. The comparison between patients with LT and hepatectomy is shown in Supplementary Table S3. Overall, the liver transplant recipients had younger age, lower tumor burden, and higher incidence of liver cirrhosis (*P* < 0.05).

**Figure 3:**
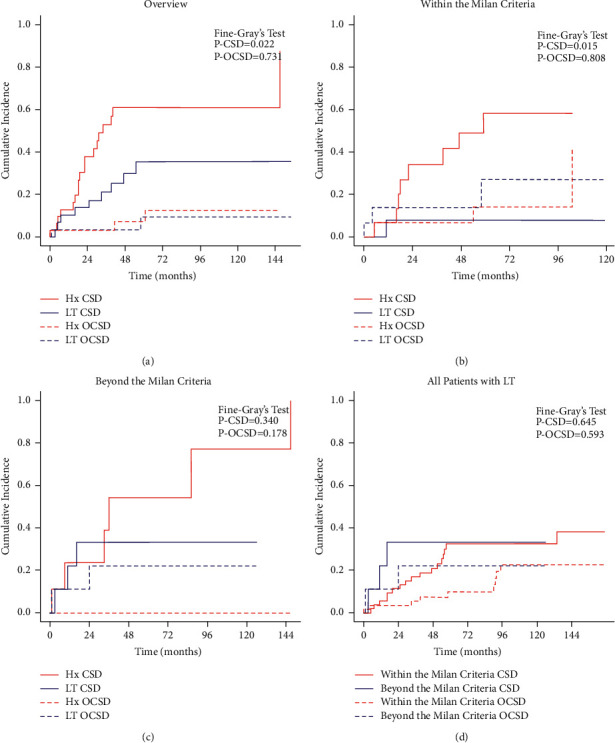
Cumulative incidence function (CIF) curves of mortality of CHC patients: (a) LT vs. Hx in all study patients; (b) LT vs. Hx in patients within the Milan Criteria; (c) LT vs. Hx in patients beyond the Milan Criteria; (d) LT in patients within and beyond the Milan Criteria. Hx hepatectomy; LT liver transplantation; CSD cancer-specific death; OCSD other cause-specific death.

After PSM (32 patients in each group), patients with LT showed better survival than those with hepatectomy (*P*=0.022, Figure 3(a)). The 1 yr, 3 yr, and 5 yr cumulative incidence of CSD were 12.8%, 52.7%, and 60.9% in the hepatectomy group and 10.2%, 21.1%, and 35.4% in the LT group separately. Then, subgroup analyses were conducted according to the Milan Criteria. Among patients within the Milan Criteria, LT could still bring survival benefits after PSM (*P*=0.015, 15 patients in each group, Supplementary Table S4 and Figure 3(b)). However, there was no survival difference between LT and hepatectomy in patients beyond the Milan Criteria after PSM (*P*=0.340, nine patients in each group, Supplementary Table S5 and Figure 3(c)). A horizontal comparison of LT outcomes of patients within and beyond the Milan Criteria is displayed in Supplementary Table S6 and Figure 3(d), but no survival difference was found between the two groups (*P*=0.645).

## 4. Discussion

CHC represents a cohort of rare and heterogeneous tumors that account for 0.4%–14.2% of primary liver cancer [1–9]. At present, the specific etiology of CHC remains unknown, but the risk factors of HCC or ICC are usually considered as risk factors of CHC as well [3, 6]. The role of nonalcoholic steatohepatitis (NASH) in the occurrence and development of primary liver cancer has been confirmed in HCC and ICC [28, 29]. In a US retrospective study, about 40% of patients had Body Mass Index values over 30, indicating that NASH may be a potential driving factor of CHC [30]. Meanwhile, a Japanese national survey showed that many patients were infected with viral hepatitis (16.4% for hepatitis B and 29.0% for hepatitis C). The rate was similar to HCC but higher than ICC [1]. Therefore, different disease spectra in western and eastern centers may cause different etiology of CHC. Besides, there is still no effective predictive model for this specific hepatobiliary tumor. Due to the rarity of CHC, it is difficult for a single institute to obtain enough research cases. Under such circumstances, the SEER database has the unique advantages of large sample capacity and population-based research background. In this study, we enrolled 736 patients, described the epidemiological and clinical features, developed and published an online nomogram based on a competing risk model, and explored the role of LT in CHC patients.

The morbidity of CHC was 0.062 per 100,000 individuals in 2004 and 0.081 per 100,000 individuals in 2018. The incidence remained stable (APC = 1.0%, *P* > 0.05), suggesting that the preventive strategy did not improve significantly. Another concern is the lack of definite imaging diagnostic, which may hamper estimates of the actual prevalence of CHC, especially in patients with liver cirrhosis. Some researchers have developed novel tools based on radiomics to improve diagnosis efficiency [31, 32]. However, compared with CT or MRI, ultrasound is more economical and convenient for primary screening. An Italy team found that different vascular criteria in contrast-enhanced ultrasound could reasonably predict the nature of liver nodules, which may provide new directions for diagnosing CHC [33].

As for gender, the APC values showed no difference (1.0% vs. 1.4%), but the incidence of males was about twice that of females, indicating an apparent male dominance of CHC, which was analogous to HCC [33, 34]. This cohort also showed variation in the incidence of CHC by ethnicity. Interestingly, although it is generally considered a high-risk factor for liver cancer, the Asia-Pacific population showed a gradual decline in the incidence of CHC in this study (APC = −5.2%, *P* < 0.05). However, it was worth noting that the incidence of the Asia-Pacific population was still as high as 0.086 per 100,000 in 2018, which was slightly higher than the overall incidence (0.081 per 100,000). Moreover, multivariate competing risk analyses indicated that the Asia-Pacific race was an independent prognostic factor (SHR = 1.464, 95% CI = 1.047–2.048, *P*=0.026). In this context, more rigorous and elaborate prevention strategies should still be implemented in these people by public health departments [35, 36].

In our cohort, the cumulative incidence of OCSD was significantly higher in patients with cancer histories, multiple tumors, and unknown tumor size (all *P* < 0.05). These competing risks may mislead the conclusions drawn from conventional survival analyses, such as the Kaplan–Meier method and the standard Cox regression model. The competing risk model could assess the informative nature of censoring and the occurrence rates of a particular event, which is much more suitable for survival analyses in the present study [15]. In addition to the race, seven other features were identified as independent prognostic factors, namely, tumor size, extrahepatic invasion, vascular invasion, distant metastasis, grade, Metavir stage, and surgery. Tumor size, extrahepatic invasion, vascular invasion, and distant metastasis are recognized as essential components of the AJCC staging system of CHC and ICC.

As another important part of the AJCC staging system, LNM was identified in 85 (11.5%) patients in this study, similar to previous studies (8.6%–21.4%) [1, 12, 14, 23, 37–42]. The incidence of LNM in CHC patients was higher than that of HCC patients (3.1%–4.9%) [43–45] but lower than that of ICC patients (22.6%–45.2%) [46–49]. Some researchers remarked on the predictive significance of LNM [23, 37, 39, 42]. Nevertheless, the same finding was not obtained in our study, supported by several previous studies [14, 50, 51]. Possible reasons that can explain this contradiction are shown as follows: Firstly, due to the highly heterogeneous nature, the predominance of HCC or ICC component may affect the biological behavior of the tumor [41, 52]. Secondly, it is difficult to differentiate CHC from HCC before surgery, resulting in a limited rate of lymph node dissection and insufficient evaluation of nodal status [1, 18, 53]. Overall, LNM showed a high incidence but a low risk of CSD in CHC patients, indicating the intermediate clinical characteristics of CHC in comparison with HCC and ICC.

Tumor grade was regarded as one of the determinants of cancer-specific survival in this study (SHR = 1.615, 95% CI = 1.149–2.271, *P*=0.006). This result was following several previous studies [51, 54, 55]. Lunsford and colleagues [25] revealed significantly superior recurrence-free survival in CHC patients with well-moderately differentiated tumors compared with poorly differentiated tumors after LT. Yamashita et al. [53] and Martin et al. [21] also confirmed that the poorly differentiated tumor was a predictor of early recurrence in CHC patients, suggesting a possible correlation between tumor grade and prognosis. Like HCC, cirrhosis was observed in the majority (62.5%) of CHC patients with known Metavir stage. The liver reserve function determines the choice of treatment to a great extent, and cirrhosis has been a well-recognized risk factor of postoperative liver failure, leading to poor outcomes for CHC patients (SHR = 2.004, 95% CI = 1.145–3.510, *P*=0.015) [56, 57].

Surgery was considered as the strongest predictor in CHC patients, but the therapeutic value of LT remains a matter of debate. Traditionally, CHC patients would not be seen as LT recipients because of the ICC component and high recurrence rate (38%–100%) [16, 19, 58, 59]. In this large-scale study, outcomes after LT were superior to hepatectomy, especially in patients within the Milan Criteria (*P*=0.022 and *P*=0.015). This finding was similar to that obtained from a recent multicenter study [20]. As for patients beyond the Milan Criteria, there was no survival difference between LT and hepatectomy (*P*=0.340). Although tumor burden did not affect the outcomes in our cohort, considering the shortage of donor livers for transplantation, LT should be carefully considered in patients beyond the Milan Criteria. Some other researchers explored the application of LT in CHC patients and drew several positive conclusions in recent years as well. Martin et al. [21] confirmed the advantages of LT in highly selected patients who have cirrhosis and unresectable small tumors (≤5 cm). Ito et al. [22] conducted an observational study and conjectured that patients within the Milan Criteria could benefit from living donor liver transplantation. Jaradat et al. [23] also affirmed the positive role of LT in early-staged CHC patients in a multicenter cohort. Although Li and colleagues [16] were skeptical about LT, their meta-analysis still admitted that prognoses of patients with LT were not worse than those of patients with hepatectomy. Overall, there are only a few studies about the efficacy of LT in CHC patients to date. Still, the current findings did not support strictly deeming CHC patients as a contraindication for LT. Although our study is a high-volume study and PSM is one of the best methods to reduce selection bias, the sample size after matching was not large enough due to the rarity of CHC, which may affect the reliability of our findings. Hence, a multicenter prospective study is needed further to confirm the role of LT in CHC patients.

A nomogram is an intuitive, understandable, and user-friendly statistical tool that allows multiple factors to be considered simultaneously and visually provides a probability of a specific outcome for an individual patient [60]. On account of the multivariate competing risk analyses, we incorporated eight easily accessible clinicopathological factors (race, extrahepatic invasion, vascular invasion, tumor size, distant metastasis, grade, Metavir stage, and surgery) to develop a nomogram for predicting the cancer-specific survival in CHC patients. For further convenience of use, we provided an online tool for individualized evaluation. The nomogram showed relatively high accuracy with Harrell's C-indexes exceeding 0.700 and well-fitted calibration curves in both the training and validation sets. Besides, the nomogram also displayed better goodness of fit according to its lower AIC and BIC values. However, high prediction accuracy is not equal to a high clinical practical value. The DCA could quantify the overall benefits of the prediction models based on the threshold probability introduced to this study to examine the value of the nomogram in clinical practice [27]. The DCA confirmed the validity of the nomogram for the CSS and demonstrated that the nomogram had better clinical value than the AJCC staging system.

As far as we know, this study is the largest sample of survival analyses of CHC patients based on the competing risk model. Although our study has many merits, including large sample size, definite pathological diagnosis, and complete follow-up, some limitations still exist. Firstly, the major drawback of this study is the inherent bias of the retrospective study. Secondly, the SEER database lacks detailed clinicopathological data, which caused unknown bias and limited further subgroup analysis. Thirdly, since the main focus of this study was on the outcomes of CHC patients, we did not horizontally compare the therapeutic value of LT between CHC, ICC, and HCC patients. Last but not least, although this study preliminarily explored the role of LT in CHC patients, considering the strict indications of transplantation, nontransplant therapy should also be taken seriously. In this cohort, the majority of patients (62.5%, 75/120) with known fibrosis scores had liver cirrhosis. Improper aggressive therapy may cause further liver damage, especially in those with borderline liver function [61]. Therefore, how to make treatment strategy individualized will be the content of our next phase of research.

## 5. Conclusion

The morbidity of CHC has remained stable in recent years. CHC appears to show intermediate clinicopathological features of HCC and ICC. Race, extrahepatic invasion, vascular invasion, tumor size, distant metastasis, grade, Metavir stage, and surgery are independent predictors of cancer-specific survival in CHC patients. The constructed nomogram could predict the prognosis with good performance, meaningful to individual treatment strategies optimization. Patients with CHC should also be considered potential liver transplant recipients, especially those within the Milan Criteria, but the finding still needs more evidence to be further confirmed.

## Figures and Tables

**Table 1:** Baseline characteristics data of CHC patients.

Factors	No. of patients (*N* = 736)
Year of diagnosis	
2004–2011	333 (45.2)
2012–2018	403 (54.8)
Age	
≤60	297 (40.4)
>60	439 (59.6)
Sex	
Female	241 (32.7)
Male	495 (67.3)
Race	
White	558 (75.8)
Asia-Pacific	100 (13.6)
Black	68 (9.2)
Other	10 (1.4)
Residence	
Urban	659 (89.5)
Rural	77 (10.5)
Income^†^	
Below the median	415 (56.4)
Above the median	321 (43.6)
AFP	
Negative	151 (20.5)
Positive	324 (44.0)
Borderline/unknown	261 (35.5)
First malignant	
Yes	621 (84.4)
No	115 (15.6)
Primary tumor	
Yes	717 (97.4)
No	19 (2.6)
Neoadjuvant therapy	
Yes	18 (2.4)
No	718 (97.6)
Tumor number	
Single	693 (94.2)
Multiple	43 (5.8)
Tumor size	
≤5 cm	297 (40.4)
>5 cm	281 (38.2)
Unknown	158 (21.4)
Surgery	
None	438 (59.5)
LD	34 (4.6)
Hx	183 (24.9)
LT	79 (10.7)
Unknown	2 (0.3)
T stage	
T1a	103 (14.0)
T1b	74 (10.1)
T1NOS	19 (2.6)
T2	277 (37.6)
T3	26 (3.5)
T4	27 (3.7)
TX	210 (28.5)
N Stage	
N0	442 (60.1)
N1	85 (11.5)
NX	209 (28.4)
M stage	
M0	518 (70.4)
M1	188 (25.5)
MX	30 (4.1)
Grade^‡^	
G1-G2	159 (21.6)
G3-G4	273 (37.1)
Unknown	304 (41.3)
Metavir stage	
F0–F3	45 (6.1)
F4	75 (10.2)
Unknown	616 (83.7)

CHC combined hepatocellular-cholangiocarcinoma; AFP alpha-fetoprotein; LD local destruction; Hx hepatectomy; LT liver transplantation; NOS not otherwise specified. ^†^US Census Bureau, Real Median Household Income in the United States [MEHOINUSA672N], retrieved from FRED, Federal Reserve Bank of St. Louis, https://fred.stlouisfed.org/series/MEHOINUSA672N, June 26, 2021; ^‡^*G*1 well differentiated; *G*2 moderately differentiated; G3-4 poorly differentiated/undifferentiated.

**Table 2:** Competing risk survival analyses of CHC patients in the training set.

Factors	No. of patients (*n* = 367)	Univariable		Multivariate	
		P-CSD	P-OCSD	SHR (95%CI)	*P*
Year of diagnosis		0.171	0.778		
2004–2011	203 (55.3)			Reference	
2012–2018	164 (44.7)			0.789 (0.593–1.050)	0.100
Age		0.138	0.199		
≤60	164 (44.7)			Reference	
>60	203 (55.3)			1.043 (0.806–1.349)	0.750
Sex		0.567	0.838		
Female	112 (30.5)			Reference	
Male	255 (69.5)			0.994 (0.729–1.357)	0.970
Race		0.843	0.644		
White	277 (75.5)			Reference	
Asia-Pacific	55 (15.0)			1.464 (1.047–2.048)	0.026
Black	31 (8.4)			1.129 (0.718–1.776)	0.600
Other	4 (1.1)			0.728 (0.372–1.425)	0.350
Residence		0.129	0.149		
Urban	336 (91.6)			Reference	
Rural	31 (8.4)			1.330 (0.921–1.920)	0.130
Income^†^		0.369	0.384		
Below the median	211 (57.5)			Reference	
Above the median	156 (42.5)			0.763 (0.573–1.015)	0.064
AFP		0.980	0.320		
Negative	94 (25.6)			Reference	
Positive	177 (48.2)			0.785 (0.563–1.095)	0.150
Borderline/unknown	96 (26.2)			0.789 (0.536–1.160)	0.230
First malignant		0.009	0.001		
Yes	316 (86.1)			Reference	
No	51 (13.9)			0.815 (0.507–1.310)	0.400
Primary tumor		0.054	0.230		
Yes	358 (97.5)			Reference	
No	9 (2.5)			1.368 (0.395–4.744)	0.620
Neoadjuvant therapy		0.079	0.463		
Yes	17 (4.6)			Reference	
No	350 (95.4)			0.603 (0.321–1.134)	0.120
Tumor number		0.160	0.001		
Single	342 (93.2)			Reference	
Multiple	25 (6.8)			0.662 (0.329–1.331)	0.250
Tumor size		<0.001	0.006		
≤5 cm	168 (45.8)			Reference	
>5 cm	148 (40.3)			1.438 (1.073–1.927)	0.015
Unknown	51 (13.9)			1.337 (0.754–2.370)	0.320
Surgery		<0.001	0.068		
None	186 (50.7)			Reference	
LD	20 (5.4)			0.387 (0.200–0.749)	0.005
Hx	112 (30.5)			0.244 (0.176–0.339)	<0.001
LT	49 (13.4)			0.128 (0.067–0.244)	<0.001
Vascular invasion		0.091	0.095		
No	249 (67.8)			Reference	
Yes	118 (32.2)			1.512 (1.138–2.008)	0.004
Visceral peritoneum invasion		0.607	0.129		
No	349 (95.1)			Reference	
Yes	18 (4.9)			0.866 (0.351–2.137)	0.760
Extrahepatic invasion		<0.001	0.257		
No	348 (94.8)			Reference	
Yes	19 (5.2)			2.233 (1.3501–3.692)	0.002
Lymph node metastasis		<0.001	0.440		
No	290 (79.0)			Reference	
Yes	49 (13.4)			0.972 (0.669–1.413)	0.880
Unknown	28 (7.6)			1.178 (0.664–2.091)	0.580
Distant metastasis		<0.001	0.810		
No	285 (77.7)			Reference	
Yes	82 (22.3)			1.518 (1.076–2.143)	0.018
Grade^‡^		<0.001	0.227		
G1-G2	90 (24.5)			Reference	
G3-G4	151 (41.1)			1.615 (1.149–2.271)	0.006
Unknown	126 (34.4)			0.908 (0.622–1.325)	0.620
Metavir stage		0.272	0.146		
F0–F3	29 (7.9)			Reference	
F4	38 (10.4)			2.004 (1.145–3.510)	0.015
Unknown	300 (81.7)			1.429 (0.951–2.147)	0.086

CHC combined hepatocellular-cholangiocarcinoma; AFP alpha-fetoprotein; LD local destruction; Hx hepatectomy; LT liver transplantation; CSD cancer-specific death; OCSD other cause-specific death; SHR subdistribution hazard ratio; CI confidence interval. ^†^US Census Bureau, Real Median Household Income in the United States [MEHOINUSA672N], retrieved from FRED, Federal Reserve Bank of St. Louis, https://fred.stlouisfed.org/series/MEHOINUSA672N, June 26, 2021; ^‡^*G*1 well differentiated; *G*2 moderately differentiated; G3-4 poorly differentiated/undifferentiated.

**Table 3:** Analyses for prognostic performances of nomogram and the AJCC stage.

Models	Harrell's C-index	*P*	AIC	BIC	1 yr AUC	3 yr AUC	5 yr AUC
Training set (*n* = 367)							
Nomogram	0.790 (0.761–0.820)	Reference	2554.974	2607.370	0.857	0.880	0.901
AJCC stage	0.659 (0.619–0.699)	*P* < 0.001	2699.362	2727.307	0.760	0.779	0.748
Validation set (*n* = 157)							
Nomogram	0.736 (0.683–0.788)	Reference	884.239	886.930	0.818	0.882	0.730
AJCC stage	0.621 (0.562–0.680)	*P* < 0.001	925.789	928.480	0.661	0.707	0.670

AJCC American Joint Committee on Cancer; AIC Akaike information criterion; BIC Bayesian information criterion; AUC area under the curve.

## Data Availability

The study cohort was taken from the Surveillance, Epidemiology, and End Results (SEER) database, which is public and desensitized. Access permission to the SEER database should be requested from the US National Cancer Institute (https://seer.cancer.gov).

## Abbreviations

CHC: Combined hepatocellular-cholangiocarcinoma
HCC: Hepatocellular carcinoma
ICC: Intrahepatic cholangiocarcinoma
AJCC: American Joint Committee on Cancer
LT: Liver transplantation
SEER: Surveillance, Epidemiology, and End Results
APC: Annual percentage change
AFP: Alpha-fetoprotein
CSS: Cancer-specific survival
CSD: Cancer-specific death
OCSD: Other cause-specific death
CIF: Cumulative incidence function
PSM: Propensity score matching
AIC: Akaike information criterion
BIC: Bayesian information criterion
AUROC: Area under receiver operating curves
DCA: Decision curve analysis
CI: Confidence interval
IQR: Interquartile range
LNM: Lymph node metastasis
NASH: Nonalcoholic steatohepatitis.

## Supplementary Materials

Supplementary Materials Figure S1: stepwise extraction process from the SEER database. Figure S2: cumulative incidence function curves of mortality of CHC patients according to different clinicopathological factors. Figure S3: ROC analyses of the nomogram and the current AJCC staging system. Table S1: baseline characteristics of CHC patients in the training and validation set. Table S2: cumulative incidence of CSD and OCSD of CHC patients in the training set. Table S3–S5: comparison between Hx and LT before and after PSM in different subgroups of CHC patients. Table S6: comparison between CHC patients with LT within and beyond the Milan Criteria. .
