# Supplementary material for: Morbidity, Prognostic Factors, and Competing Risk Nomogram for Combined Hepatocellular-Cholangiocarcinoma
Source: J Oncol. 2021 Dec 10;2021:3002480. doi: 10.1155/2021/3002480 (PMC8683178; doi:10.1155/2021/3002480)
Supplement: Supplementary Materials — Figure S1: stepwise extraction process from the SEER database. Figure S2: cumulative incidence function curves of mortality of CHC patients according to different clinicopathological factors. Figure S3: ROC analyses of the nomogram and the current AJCC staging system. Table S1: baseline characteristics of CHC patients in the training and validation set. Table S2: cumulative incidence of CSD and OCSD of CHC patients in the training set. Table S3–S5: comparison between Hx and LT before and after PSM in different subgroups of CHC patients. Table S6: comparison between CHC patients with LT within and beyond the Milan Criteria. . [file 3002480.f1.docx]

**Contents**

| Figure/Table | Legends | Page |
| --- | --- | --- |
| Figure S1 | Stepwise extraction process from the Surveillance, Epidemiology, and End Results database | 2 |
| Figure S2 | Cumulative incidence function (CIF) curves of mortality of CHC patients according to **(a)** Year of Diagnosis, **(b)** Age, **(c)** Sex, **(d)** Race, **(e)** Residence, **(f)** Income, **(g)** AFP, **(h)** First Malignant, **(i)** Primary Tumor, **(j)** Neoadjuvant Therapy, **(k)** Tumor Number, **(l)** Tumor Size, **(m)** Surgery, **(n)** Vascular Invasion, **(o)** Visceral Peritoneum Invasion, **(p)** Extrahepatic Invasion, **(q)** Lymph Node Metastasis, **(r)** Distant Metastasis, **(s)** Grade and **(t)** METAVIR Stage. CHC=Combined hepatocellular and cholangiocarcinoma; AFP=Alpha fetoprotein; LD=Local destruction; Hx=Hepatectomy; LT=Liver transplantation; CSD=Cancer-specific death; OCSD=Other cause-specific death. | 3 |
| Figure S3 | Receiver operative characteristics (ROC) analyses of the nomogram and the current AJCC staging system (8^th^ edition) in the prediction of prognosis of patients at 1-, 3-, 5- year point for cancer-specific survival (CSS). **(a)**-**(c)** Training set; **(d)**-**(f)** Validation set. AJCC= American Joint Committee on Cancer; AUC=Area under the curve. | 4 |
| Table S1 | Baseline characteristics of CHC patients in the training and validation set | 5 |
| Table S2 | Cumulative incidence of CSD and OCSD of CHC patients in the training set | 7 |
| Table S3 | Comparison between Hx and LT before and after PSM in all CHC patients | 9 |
| Table S4 | Comparison between Hx and LT before and after PSM in CHC patients within the Milan Criteria | 11 |
| Table S5 | Comparison between Hx and LT before and after PSM in CHC patients beyond the Milan Criteria | 13 |
| Table S6 | Comparison between CHC patients with LT within and beyond the Milan Criteria | 15 |

**Figure S1**. Stepwise extraction process from the Surveillance, Epidemiology, and End Results database.

**Figure S2**. Cumulative incidence function (CIF) curves of mortality of CHC patients according to **(a)** Year of Diagnosis, **(b)** Age, **(c)** Sex, **(d)** Race, **(e)** Residence, **(f)** Income, **(g)** AFP, **(h)** First Malignant, **(i)** Primary Tumor, **(j)** Neoadjuvant Therapy, **(k)** Tumor Number, **(l)** Tumor Size, **(m)** Surgery, **(n)** Vascular Invasion, **(o)** Visceral Peritoneum Invasion, **(p)** Extrahepatic Invasion, **(q)** Lymph Node Metastasis, **(r)** Distant Metastasis, **(s)** Grade and **(t)** METAVIR Stage. CHC=Combined hepatocellular and cholangiocarcinoma; AFP=Alpha fetoprotein; LD=Local destruction; Hx=Hepatectomy; LT=Liver transplantation; CSD=Cancer-specific death; OCSD=Other cause-specific death.


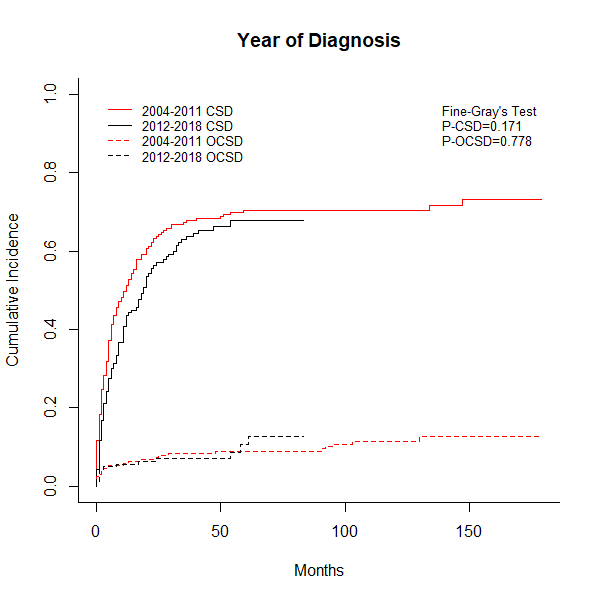

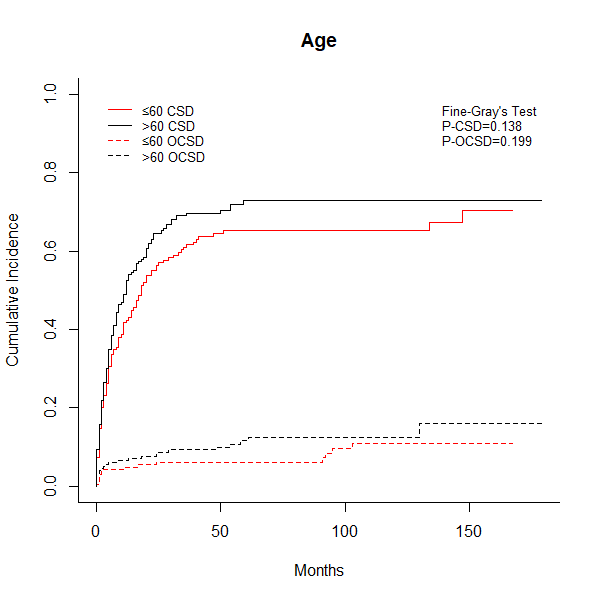

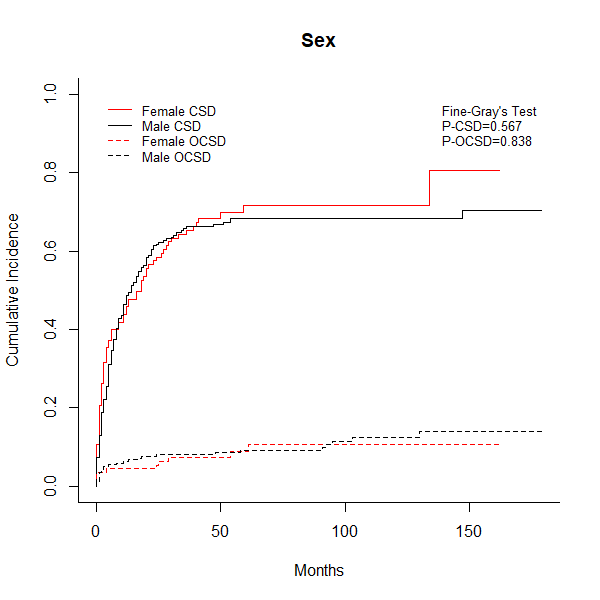

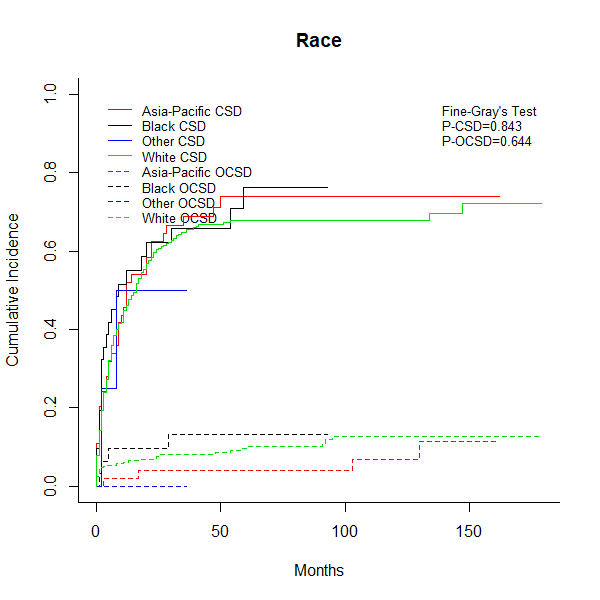

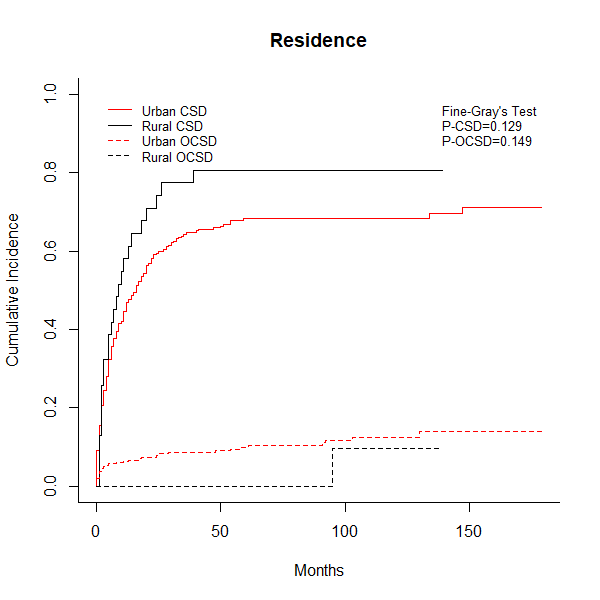

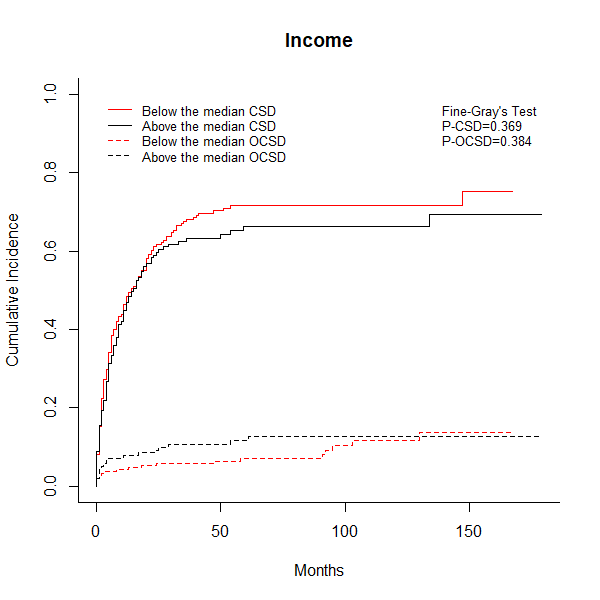

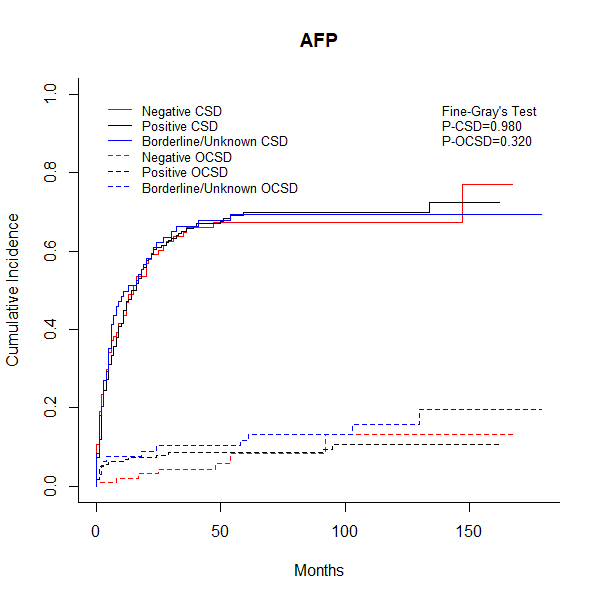

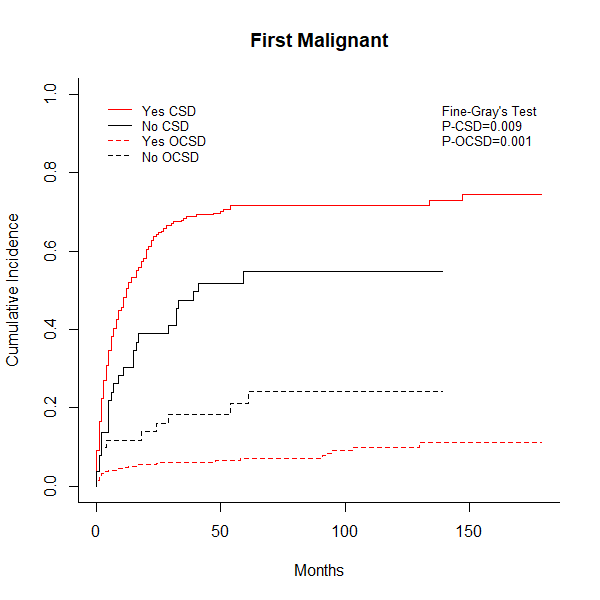

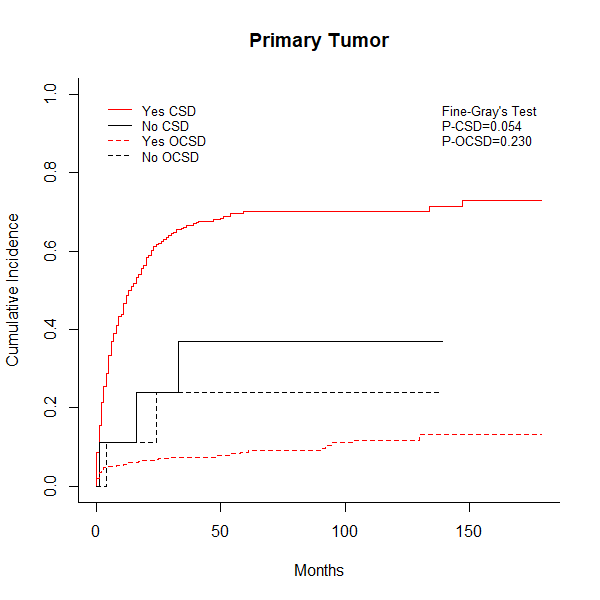

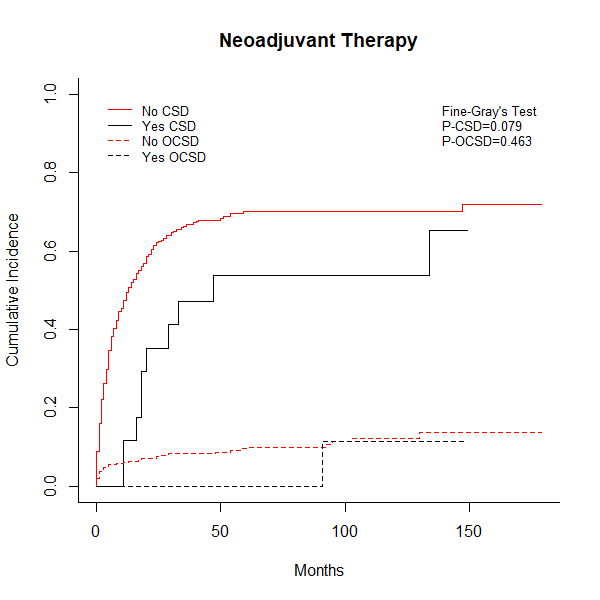

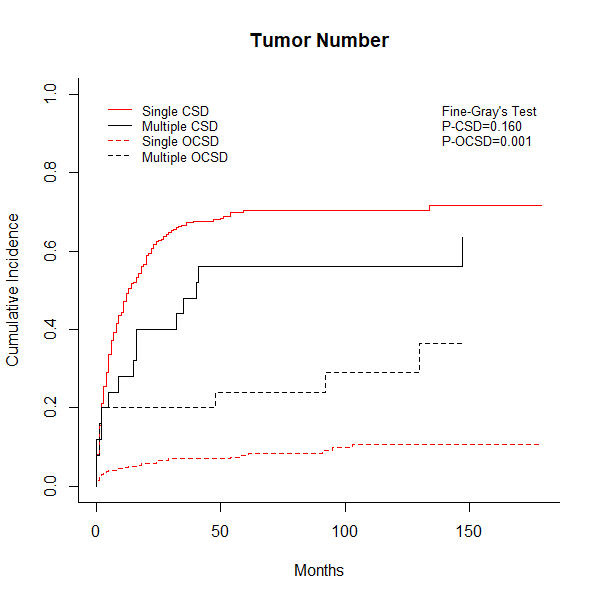

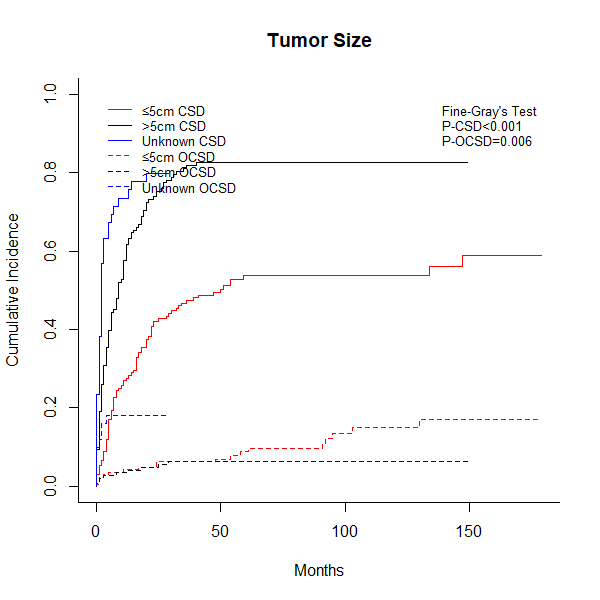

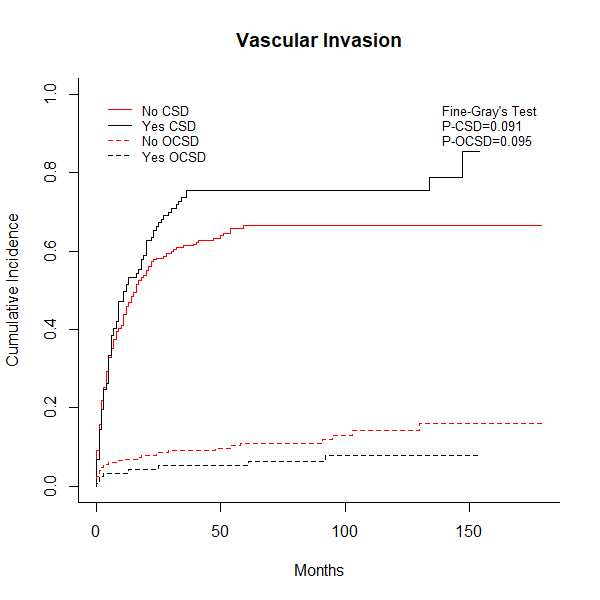

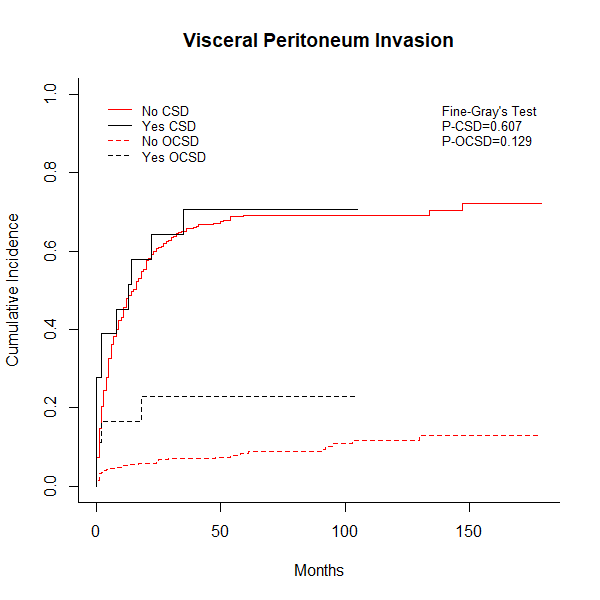

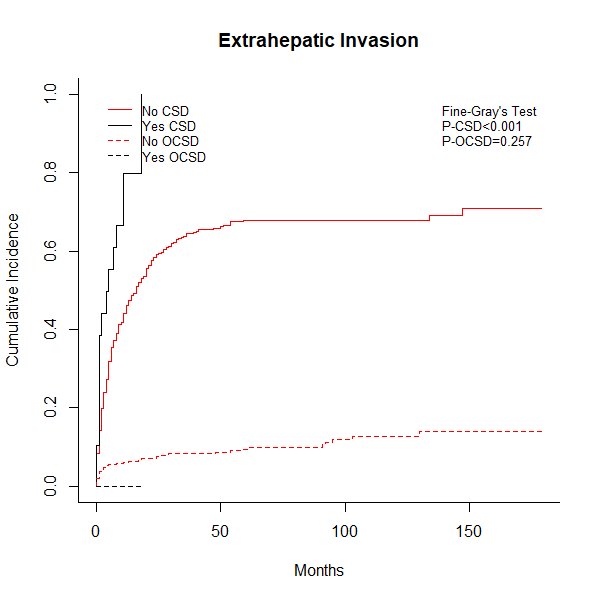

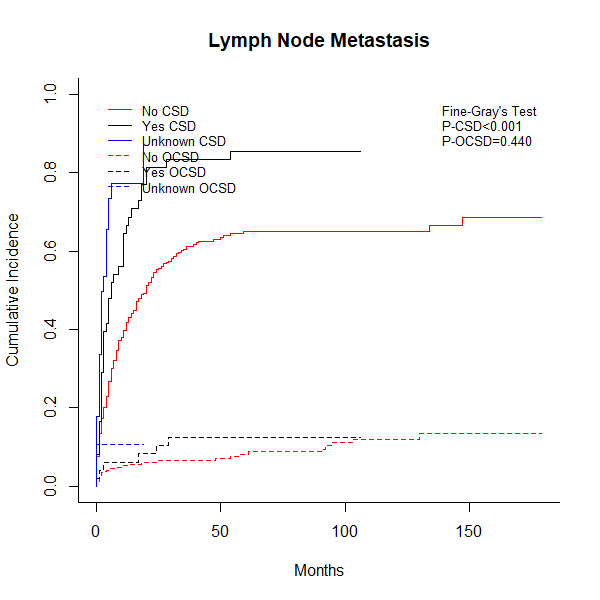

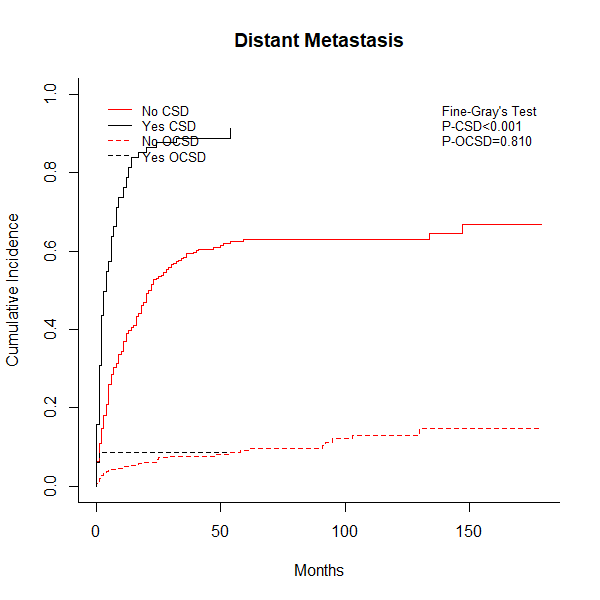

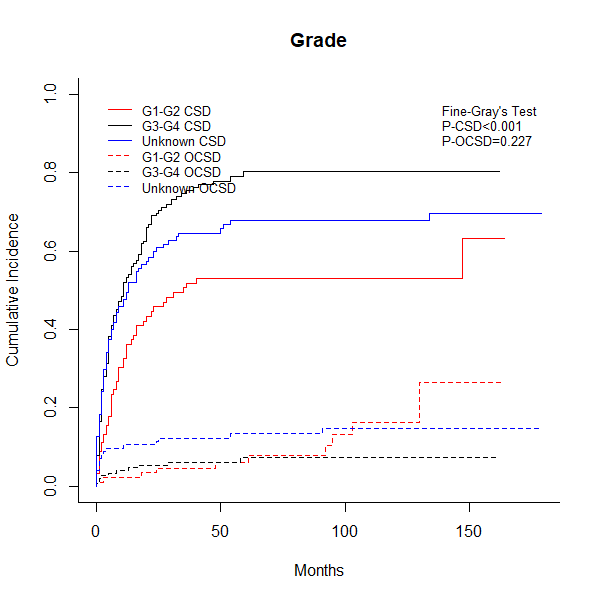

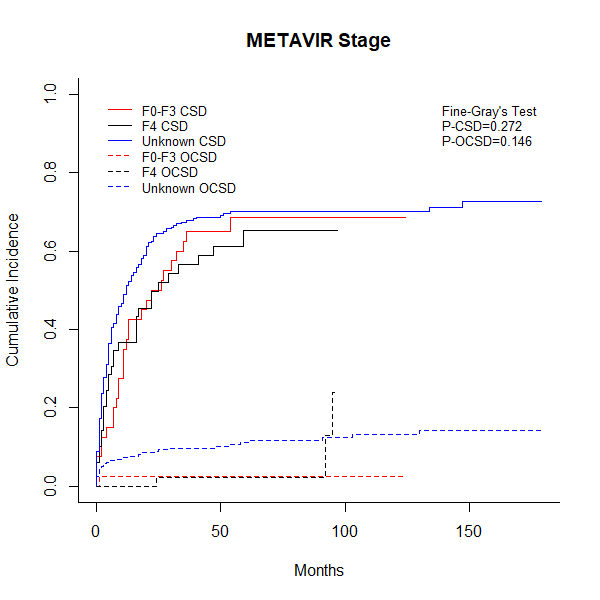

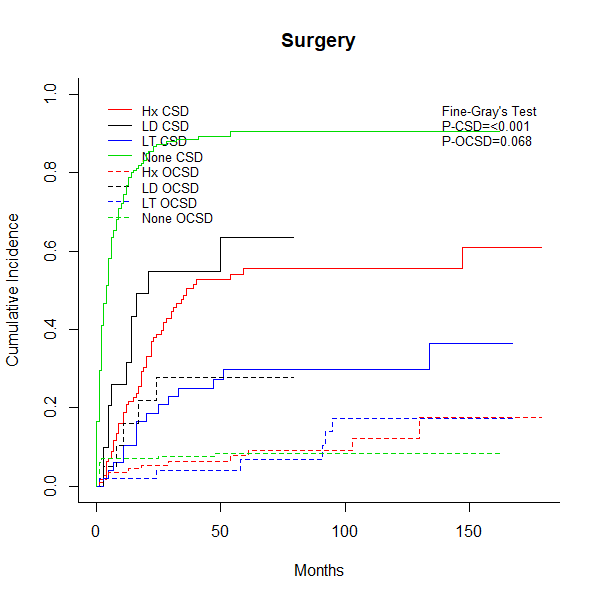


**(a)**

**(b)**

**(c)**

**(d)**

**(e)**

**(f)**

**(g)**

**(h)**

**(i)**

**(j)**

**(k)**

**(l)**

**(m)**

**(n)**

**(o)**

**(p)**

**(q)**

**(r)**

**(s)**

**(t)**


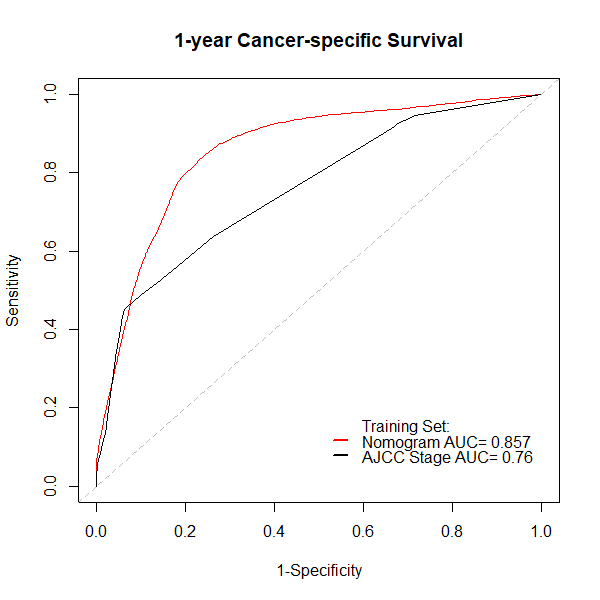

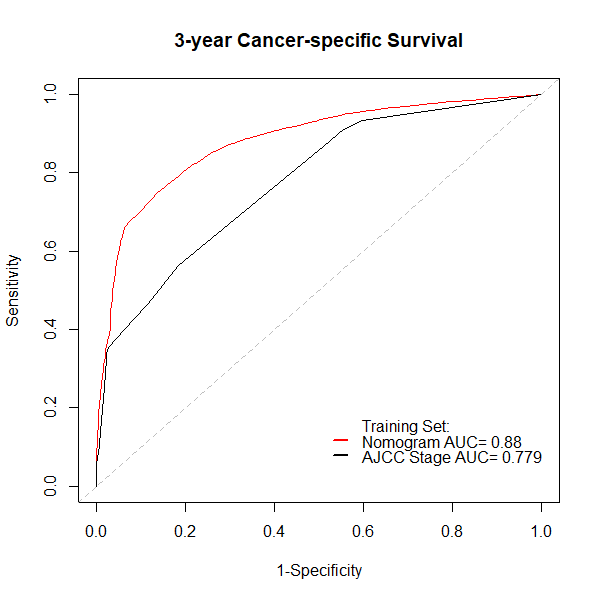

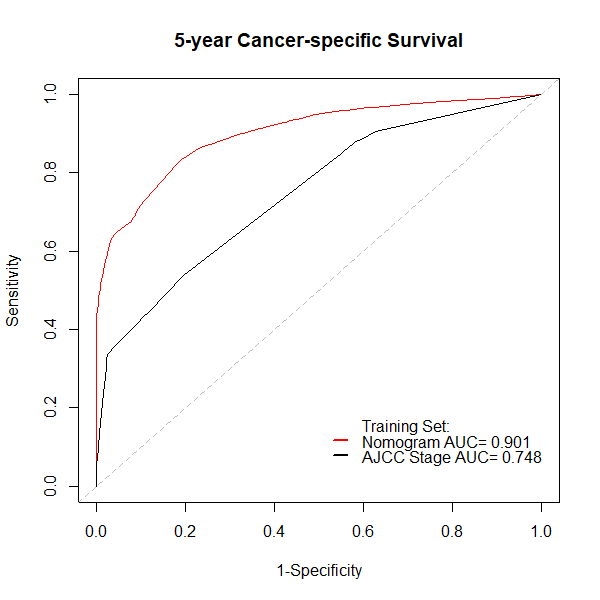

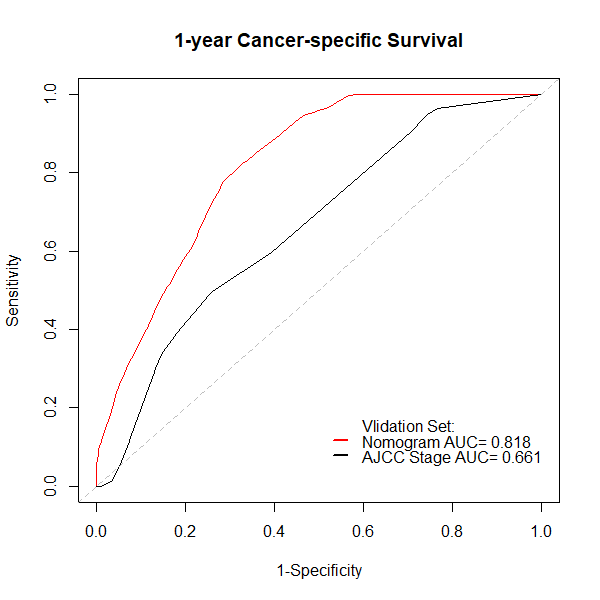

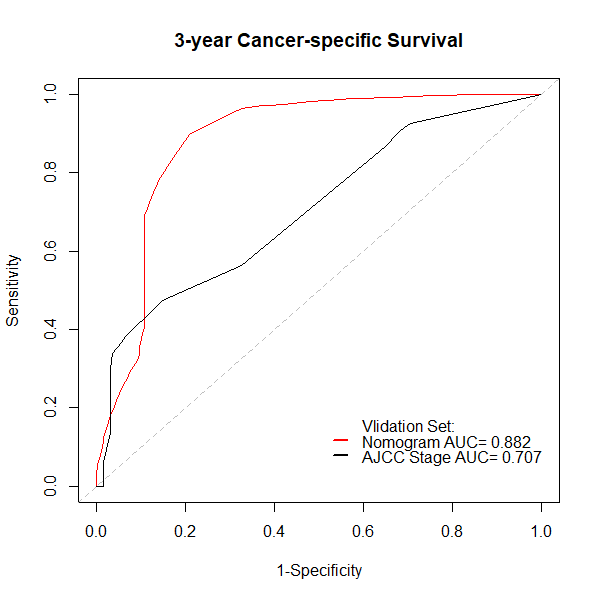

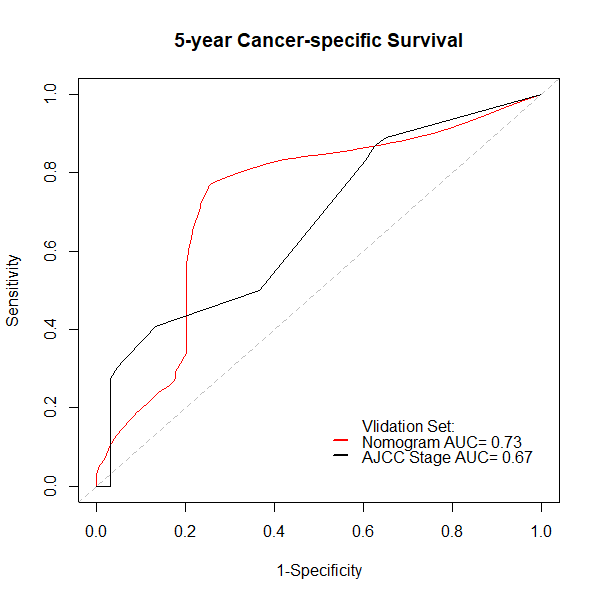


**(a)**

**(b)**

**(c)**

**(d)**

**(e)**

**(f)**

**Figure S3**. Receiver operative characteristics (ROC) analyses of the nomogram and the current AJCC staging system (8^th^ edition) in the prediction of prognosis of patients at 1-, 3-, 5- year point for cancer-specific survival (CSS). **(a)**-**(c)** Training set; **(d)**-**(f)** Validation set. AJCC= American Joint Committee on Cancer; AUC=Area under the curve.

**Table S1**. Baseline characteristics of CHC patients in the training and validation set

| Factors | All Patients  (n=524) | Training Set  (n=367) | Validation Set  (n=157) | P |
| --- | --- | --- | --- | --- |
| Year of Diagnosis |  |  |  | 0.876 |
| 2004-2011 | 291(55.5) | 203(55.3) | 88(56.1) |  |
| 2012-2018 | 233(44.5) | 164(44.7) | 69(43.9) |  |
| Age |  |  |  | 0.671 |
| ≤60 | 231(44.1) | 164(44.7) | 67(42.7) |  |
| >60 | 293(55.9) | 203(55.3) | 90(57.3) |  |
| Sex |  |  |  | 0.556 |
| Female | 164(31.3) | 112(30.5) | 52(33.1) |  |
| Male | 360(68.7) | 255(69.5) | 105(66.9) |  |
| Race |  |  |  | 0.727 |
| White | 388(74.0) | 277(75.5) | 111(70.7) |  |
| Asia-Pacific | 83(15.8) | 55(15.0) | 28(17.8) |  |
| Black | 47(9.0) | 31(8.4) | 16(10.2) |  |
| Other | 6(1.2) | 4(1.1) | 2(1.3) |  |
| Residence |  |  |  | 0.277 |
| Urban | 475(90.6) | 336(91.6) | 139(88.5) |  |
| Rural | 49(9.4) | 31(8.4) | 18(11.5) |  |
| Income^†^ |  |  |  | 0.711 |
| Below the median | 304(58.0) | 211(57.5) | 93(59.2) |  |
| Above the median | 220(42.0) | 156(42.5) | 64(40.8) |  |
| AFP |  |  |  | 0.025 |
| Negative | 120(22.9) | 94(25.6) | 26(16.6) |  |
| Positive | 252(48.1) | 177(48.2) | 75(47.8) |  |
| Borderline/Unknown | 152(29.0) | 96(26.2) | 56(35.7) |  |
| First Malignant |  |  |  | 0.248 |
| Yes | 445(84.9) | 316(86.1) | 129(82.2) |  |
| No | 79(15.1) | 51(13.9) | 28(17.8) |  |
| Primary Tumor |  |  |  | 0.565 |
| Yes | 509(97.1) | 358(97.5) | 151(96.2) |  |
| No | 15(2.9) | 9(2.5) | 6(3.8) |  |
| Neoadjuvant Therapy |  |  |  | 0.021 |
| Yes | 18(3.4) | 17(4.6) | 1(0.6) |  |
| No | 506(96.6) | 350(95.4) | 156(99.4) |  |
| Tumor Number |  |  |  | 0.279 |
| Single | 484(92.4) | 342(93.2) | 142(90.4) |  |
| Multiple | 40(7.6) | 25(6.8) | 15(9.6) |  |
| Tumor Size |  |  |  | 0.447 |
| ≤5cm | 233(44.5) | 168(45.8) | 65(41.4) |  |
| >5cm | 212(40.5) | 148(40.3) | 64(40.8) |  |
| Unknown | 79(15.1) | 51(13.9) | 28(17.8) |  |
| Surgery |  |  |  | 0.166 |
| None | 282(53.8) | 186(50.7) | 96(61.1) |  |
| LD | 26(5.0) | 20(5.4) | 6(3.8) |  |
| Hx | 152(29.0) | 112(30.5) | 40(25.5) |  |
| LT | 64(12.2) | 49(13.4) | 15(9.6) |  |
| Vascular Invasion |  |  |  | 0.168 |
| No | 365(69.7) | 249(67.8) | 116(73.9) |  |
| Yes | 159(30.3) | 118(32.2) | 41(26.1) |  |
| Visceral Peritoneum Invasion |  |  |  | 0.927 |
| No | 498(95.0) | 349(95.1) | 149(94.9) |  |
| Yes | 26(5.0) | 18(4.9) | 8(5.1) |  |
| Extrahepatic Invasion |  |  |  | 0.505 |
| No | 499(95.2) | 348(94.8) | 151(96.2) |  |
| Yes | 25(4.8) | 19(5.2) | 6(3.8) |  |
| Lymph Node Metastasis |  |  |  | 0.905 |
| No | 413(78.8) | 290(79.0) | 123(78.3) |  |
| Yes | 72(13.7) | 49(13.4) | 23(14.6) |  |
| Unknown | 39(7.4) | 28(7.6) | 11(7.1) |  |
| Distant Metastasis |  |  |  | 0.862 |
| No | 408(77.9) | 285(77.7) | 123(78.3) |  |
| Yes | 116(22.1) | 82(22.3) | 34(21.7) |  |
| Grade^‡^ |  |  |  | 0.138 |
| G1-G2 | 130(24.8) | 90(24.5) | 40(25.5) |  |
| G3-G4 | 202(38.5) | 151(41.1) | 51(32.5) |  |
| Unknown | 192(36.7) | 126(34.4) | 66(42.0) |  |
| Metavir Stage |  |  |  | 0.210 |
| F0-F3 | 38(7.3) | 29(7.9) | 9(5.7) |  |
| F4 | 48(9.2) | 38(10.4) | 10(6.4) |  |
| Unknown | 438(83.5) | 300(81.7) | 138(87.9) |  |

CHC=Combined hepatocellular-cholangiocarcinoma; AFP=Alpha fetoprotein; LD=Local destruction; Hx=Hepatectomy; LT=Liver transplantation.

^†^U.S. Census Bureau, Real Median Household Income in the United States [MEHOINUSA672N], retrieved from FRED, Federal Reserve Bank of St. Louis; https://fred.stlouisfed.org/series/MEHOINUSA672N, June 26, 2021

^‡^G1=Well differentiated; G2=Moderately differentiated; G3-4=Poorly differentiated/Undifferentiated.

**Table S2**. Cumulative incidence of CSD and OCSD of CHC patients in the training set

| Factors | CSD | | | OCSD | | |
| --- | --- | --- | --- | --- | --- | --- |
|  | 1yr-CI | 3yr-CI | 5yr-CI | 1yr-CI | 3yr-CI | 5yr-CI |
| Year of Diagnosis |  |  |  |  |  |  |
| 2004-2011 | 0.512 | 0.678 | 0.705 | 0.060 | 0.085 | 0.090 |
| 2012-2018 | 0.436 | 0.638 | 0.678 | 0.056 | 0.070 | 0.106 |
| Age |  |  |  |  |  |  |
| ≤60 | 0.424 | 0.616 | 0.652 | 0.049 | 0.062 | 0.062 |
| >60 | 0.524 | 0.697 | 0.729 | 0.065 | 0.093 | 0.116 |
| Sex |  |  |  |  |  |  |
| Female | 0.458 | 0.653 | 0.716 | 0.045 | 0.074 | 0.089 |
| Male | 0.486 | 0.662 | 0.684 | 0.064 | 0.081 | 0.091 |
| Race |  |  |  |  |  |  |
| White | 0.461 | 0.655 | 0.678 | 0.062 | 0.081 | 0.097 |
| Asia-Pacific | 0.520 | 0.688 | 0.740 | 0.019 | 0.040 | 0.040 |
| Black | 0.551 | 0.657 | 0.762 | 0.097 | 0.132 | 0.132 |
| Other | 0.500 | 0.500 | - | 0 | 0 | - |
| Residence |  |  |  |  |  |  |
| Urban | 0.468 | 0.649 | 0.682 | 0.064 | 0.086 | 0.100 |
| Rural | 0.581 | 0.774 | 0.806 | 0 | 0 | 0 |
| Income^†^ |  |  |  |  |  |  |
| Below the median | 0.484 | 0.680 | 0.716 | 0.043 | 0.058 | 0.071 |
| Above the median | 0.470 | 0.632 | 0.664 | 0.079 | 0.107 | 0.117 |
| AFP |  |  |  |  |  |  |
| Negative | 0.469 | 0.660 | 0.673 | 0.021 | 0.044 | 0.083 |
| Positive | 0.472 | 0.657 | 0.699 | 0.068 | 0.086 | 0.086 |
| Borderline/Unknown | 0.498 | 0.663 | 0.692 | 0.076 | 0.103 | 0.118 |
| First Malignant |  |  |  |  |  |  |
| Yes | 0.506 | 0.689 | 0.716 | 0.048 | 0.062 | 0.071 |
| No | 0.303 | 0.475 | 0.548 | 0.118 | 0.182 | 0.212 |
| Primary Tumor |  |  |  |  |  |  |
| Yes | 0.487 | 0.667 | 0.701 | 0.057 | 0.075 | 0.087 |
| No | 0.111 | 0.370 | 0.370 | 0.111 | 0.241 | 0.241 |
| Neoadjuvant Therapy |  |  |  |  |  |  |
| Yes | 0.118 | 0.471 | 0.537 | 0 | 0 | 0 |
| No | 0.469 | 0.669 | 0.701 | 0.061 | 0.083 | 0.096 |
| Tumor Number |  |  |  |  |  |  |
| Single | 0.493 | 0.673 | 0.703 | 0.048 | 0.070 | 0.080 |
| Multiple | 0.280 | 0.480 | 0.560 | 0.200 | 0.200 | 0.240 |
| Tumor Size |  |  |  |  |  |  |
| ≤5cm | 0.276 | 0.474 | 0.538 | 0.043 | 0.062 | 0.088 |
| >5cm | 0.618 | 0.819 | 0.827 | 0.034 | 0.063 | 0.063 |
| Unknown | 0.735 | - | - | 0.181 | - | - |
| Surgery |  |  |  |  |  |  |
| None | 0.769 | 0.886 | 0.904 | 0.071 | 0.077 | 0.084 |
| LD | 0.317 | 0.549 | 0.636 | 0.161 | 0.277 | 0.277 |
| Hx | 0.207 | 0.505 | 0.555 | 0.036 | 0.064 | 0.078 |
| LT | 0.104 | 0.250 | 0.298 | 0.020 | 0.041 | 0.069 |
| Vascular Invasion |  |  |  |  |  |  |
| No | 0.459 | 0.614 | 0.665 | 0.070 | 0.092 | 0.110 |
| Yes | 0.516 | 0.755 | 0.755 | 0.034 | 0.052 | 0.052 |
| Visceral Peritoneum Invasion |  |  |  |  |  |  |
| No | 0.479 | 0.657 | 0.692 | 0.052 | 0.071 | 0.084 |
| Yes | 0.452 | 0.706 | 0.706 | 0.167 | 0.230 | 0.230 |
| Extrahepatic Invasion |  |  |  |  |  |  |
| No | 0.462 | 0.645 | 0.679 | 0.061 | 0.083 | 0.095 |
| Yes | 0.799 | - | - | 0 | - | - |
| Lymph Node Metastasis |  |  |  |  |  |  |
| No | 0.419 | 0.613 | 0.650 | 0.052 | 0.067 | 0.082 |
| Yes | 0.666 | 0.833 | 0.854 | 0.062 | 0.125 | 0.125 |
| Unknown | 0.774 | - | - | 0.107 | - | - |
| Distant Metastasis |  |  |  |  |  |  |
| No | 0.389 | 0.593 | 0.630 | 0.050 | 0.077 | 0.092 |
| Yes | 0.788 | 0.889 | - | 0.086 | 0.086 | - |
| Grade^‡^ |  |  |  |  |  |  |
| G1-G2 | 0.362 | 0.518 | 0.530 | 0.022 | 0.046 | 0.060 |
| G3-G4 | 0.534 | 0.755 | 0.803 | 0.040 | 0.062 | 0.074 |
| Unknown | 0.494 | 0.646 | 0.679 | 0.106 | 0.123 | 0.134 |
| Metavir Stage |  |  |  |  |  |  |
| F0-F3 | 0.375 | 0.650 | 0.686 | 0.025 | 0.025 | 0.025 |
| F4 | 0.367 | 0.565 | 0.653 | 0 | 0.022 | 0.022 |
| Unknown | 0.514 | 0.678 | 0.701 | 0.073 | 0.098 | 0.113 |

CSD=Cancer-specific death; OCSD=Other cause-specific death; CHC=Combined hepatocellular-cholangiocarcinoma; CI=Confidence interval; AFP=Alpha fetoprotein; LD=Local destruction; Hx=Hepatectomy; LT=Liver transplantation;

^†^U.S. Census Bureau, Real Median Household Income in the United States [MEHOINUSA672N], retrieved from FRED, Federal Reserve Bank of St. Louis; https://fred.stlouisfed.org/series/MEHOINUSA672N, June 26, 2021

^‡^G1=Well differentiated; G2=Moderately differentiated; G3-4=Poorly differentiated/Undifferentiated.

**Table S3**. Comparison between Hx and LT before and after PSM in all CHC patients

| Factors | Before PSM | | | After PSM | | |
| --- | --- | --- | --- | --- | --- | --- |
|  | Hx  (n=183) | LT  (n=79) | P | Hx  (n=32) | LT  (n=32) | P |
| Year of Diagnosis |  |  | 0.096 |  |  | 1.000 |
| 2004-2011 | 73(39.9) | 41(51.9) |  | 13(40.6) | 14(43.8) |  |
| 2012-2018 | 110(60.1) | 38(48.1) |  | 19(59.4) | 18(56.2) |  |
| Age |  |  | <0.001 |  |  | 1.000 |
| ≤60 | 74(40.4) | 54(68.4) |  | 17(53.1) | 17(53.1) |  |
| >60 | 109(59.6) | 25(31.6) |  | 15(46.9) | 15(46.9) |  |
| Sex |  |  | 0.129 |  |  | 1.000 |
| Female | 63(34.4) | 19(24.1) |  | 11(34.4) | 10(31.2) |  |
| Male | 120(65.6) | 60(75.9) |  | 21(65.6) | 22(68.8) |  |
| Race |  |  | 0.006 |  |  | 0.759 |
| White | 124(67.8) | 69(87.3) |  | 28(87.5) | 26(81.2) |  |
| Asia-Pacific | 41(22.4) | 5(6.3) |  | 3(9.4) | 4(12.5) |  |
| Black | 15(8.2) | 5(6.3) |  | 1(3.1) | 2(6.2) |  |
| Other | 3(1.6) | 0(0) |  | 0(0) | 0(0) |  |
| Residence |  |  | 1.000 |  |  | 1.000 |
| Urban | 166(90.7) | 72(91.1) |  | 29(90.6) | 29(90.6) |  |
| Rural | 17(9.3) | 7(8.9) |  | 3(9.4) | 3(9.4) |  |
| Income^†^ |  |  | 1.000 |  |  | 0.802 |
| Below the median | 101(55.2) | 43(54.4) |  | 18(56.3) | 16(50.0) |  |
| Above the median | 82(44.8) | 36(45.6) |  | 14(43.8) | 16(50.0) |  |
| AFP |  |  | 0.702 |  |  | 0.576 |
| Negative | 43(23.5) | 19(24.1) |  | 8(25.0) | 9(28.1) |  |
| Positive | 79(43.2) | 30(38.0) |  | 9(28.1) | 12(37.5) |  |
| Borderline/Unknown | 61(33.3) | 30(38.0) |  | 15(46.9) | 11(34.4) |  |
| First Malignant |  |  | 0.539 |  |  | 1.000 |
| Yes | 149(81.4) | 61(77.2) |  | 25(78.1) | 24(75.0) |  |
| No | 34(18.6) | 18(22.8) |  | 7(21.9) | 8(25.0) |  |
| Primary Tumor |  |  | 0.010 |  |  | 0.668 |
| Yes | 178(97.3) | 70(88.6) |  | 30(93.8) | 28(87.5) |  |
| No | 5(2.7) | 9(11.4) |  | 2(6.2) | 4(12.5) |  |
| Neoadjuvant Therapy |  |  | <0.001 |  |  | 1.000 |
| Yes | 4(2.2) | 14(17.7) |  | 2(6.2) | 3(9.4) |  |
| No | 179(97.8) | 65(82.3) |  | 30(93.8) | 29(90.6) |  |
| Tumor Number |  |  | 0.969 |  |  | 1.000 |
| Single | 171(93.4) | 73(92.4) |  | 28(87.5) | 28(87.5) |  |
| Multiple | 12(6.6) | 6(7.6) |  | 4(12.5) | 4(12.5) |  |
| Tumor Size |  |  | <0.001 |  |  | 0.608 |
| ≤5cm | 89(48.6) | 72(91.1) |  | 24(75.0) | 27(84.4) |  |
| >5cm | 90(49.2) | 5(6.3) |  | 7(21.9) | 4(12.5) |  |
| Unknown | 4(2.2) | 2(2.5) |  | 1(3.1) | 1(3.1) |  |
| T Stage |  |  | 0.145 |  |  | 0.861 |
| T1 | 65(35.5) | 27(34.2) |  | 12(37.5) | 14(43.8) |  |
| T2 | 73(39.9) | 37(46.8) |  | 15(46.9) | 14(43.8) |  |
| T3 | 6(3.3) | 0(0) |  | 0(0) | 0(0) |  |
| T4 | 8(4.4) | 0(0) |  | 0(0) | 0(0) |  |
| TX | 31(16.9) | 15(19.0) |  | 5(15.6) | 4(12.5) |  |
| N Stage |  |  | 0.213 |  |  | 0.937 |
| N0 | 141(77.0) | 63(79.7) |  | 26(81.2) | 27(84.4) |  |
| N1 | 15(8.2) | 2(2.5) |  | 1(3.1) | 1(3.1) |  |
| NX | 27(14.8) | 14(17.7) |  | 5(15.6) | 4(12.5) |  |
| M Stage |  |  | 0.526 |  |  | 1.000 |
| M0 | 173(94.5) | 77(97.5) |  | 31(96.9) | 32(100.0) |  |
| M1 | 7(3.8) | 1(1.3) |  | 0(0) | 0(0) |  |
| MX | 3(1.6) | 1(1.3) |  | 1(3.1) | 0(0) |  |
| Grade^‡^ |  |  | <0.001 |  |  | 0.961 |
| G1-G2 | 59(32.2) | 39(49.4) |  | 16(50.0) | 15(46.9) |  |
| G3-G4 | 92(50.3) | 15(19.0) |  | 10(31.2) | 11(34.4) |  |
| Unknown | 32(17.5) | 25(31.6) |  | 6(18.8) | 6(18.8) |  |
| Metavir Stage |  |  | <0.001 |  |  | 0.117 |
| F0-F3 | 30(16.4) | 1(1.3) |  | 0(0) | 1(3.1) |  |
| F4 | 21(11.5) | 20(25.3) |  | 5(15.6) | 11(34.4) |  |
| Unknown | 132(72.1) | 58(73.4) |  | 27(84.4) | 20(62.5) |  |
| Milan Criteria |  |  | <0.001 |  |  | 0.658 |
| Within | 66(36.1) | 55(69.6) |  | 20(62.5) | 22(68.8) |  |
| Beyond | 102(55.7) | 9(11.4) |  | 9(28.1) | 6(18.8) |  |
| Unknown | 15(8.2) | 15(19.0) |  | 3(9.4) | 4(12.5) |  |

Hx=Hepatectomy; LT=Liver transplantation; PSM=Propensity score matching; CHC=Combined hepatocellular-cholangiocarcinoma; AFP=Alpha fetoprotein.

^†^U.S. Census Bureau, Real Median Household Income in the United States [MEHOINUSA672N], retrieved from FRED, Federal Reserve Bank of St. Louis; https://fred.stlouisfed.org/series/MEHOINUSA672N, June 26, 2021

^‡^G1=Well differentiated; G2=Moderately differentiated; G3-4=Poorly differentiated/Undifferentiated.

**Table S4**. Comparison between Hx and LT before and after PSM in CHC patients within the Milan Criteria

| Factors | Before PSM | | | After PSM | | |
| --- | --- | --- | --- | --- | --- | --- |
|  | Hx  (n=66) | LT  (n=55) | P | Hx  (n=15) | LT  (n=15) | P |
| Year of Diagnosis |  |  | 0.070 |  |  | 0.714 |
| 2004-2011 | 30(45.5) | 35(63.6) |  | 8(53.3) | 6(40.0) |  |
| 2012-2018 | 36(54.5) | 20(36.4) |  | 7(46.7) | 9(60.0) |  |
| Age |  |  | 0.001 |  |  | 1.000 |
| ≤60 | 25(37.9) | 38(69.1) |  | 7(46.7) | 7(46.7) |  |
| >60 | 41(62.1) | 17(30.9) |  | 8(53.3) | 8(53.3) |  |
| Sex |  |  | 0.427 |  |  | 0.699 |
| Female | 21(31.8) | 13(23.6) |  | 6(40.0) | 4(26.7) |  |
| Male | 45(68.2) | 42(76.4) |  | 9(60.0) | 11(73.3) |  |
| Race |  |  | 0.026 |  |  | 0.502 |
| White | 40(60.6) | 46(83.6) |  | 11(73.3) | 12(80.0) |  |
| Asia-Pacific | 17(25.8) | 4(7.3) |  | 1(6.7) | 2(13.3) |  |
| Black | 8(12.1) | 5(9.1) |  | 3(20.0) | 1(6.7) |  |
| Other | 1(1.5) | 0(0) |  | 0(0) | 0(0) |  |
| Residence |  |  | 0.327 |  |  | 1.000 |
| Urban | 63(95.5) | 49(89.1) |  | 14(93.3) | 13(86.7) |  |
| Rural | 3(4.5) | 6(10.9) |  | 1(6.7) | 2(13.3) |  |
| Income^†^ |  |  | 1.000 |  |  | 1.000 |
| Below the median | 35(53.0) | 30(54.5) |  | 7(46.7) | 7(46.7) |  |
| Above the median | 31(47.0) | 25(45.5) |  | 8(53.3) | 8(53.3) |  |
| AFP |  |  | 0.254 |  |  | 0.740 |
| Negative | 15(22.7) | 18(32.7) |  | 5(33.3) | 4(26.7) |  |
| Positive | 27(40.9) | 24(43.6) |  | 4(26.7) | 6(40.0) |  |
| Borderline/Unknown | 24(36.4) | 13(23.6) |  | 6(40.0) | 5(33.3) |  |
| First Malignant |  |  | 0.590 |  |  | 1.000 |
| Yes | 53(80.3) | 41(74.5) |  | 11(73.3) | 12(80.0) |  |
| No | 13(19.7) | 14(25.5) |  | 4(26.7) | 3(20.0) |  |
| Primary Tumor |  |  | 0.195 |  |  | - |
| Yes | 63(95.5) | 48(87.3) |  | 15(100.0) | 15(100.0) |  |
| No | 3(4.5) | 7(12.7) |  | 0(0) | 0(0) |  |
| Neoadjuvant Therapy |  |  | 0.001 |  |  | 1.000 |
| Yes | 1(1.5) | 12(21.8) |  | 1(6.7) | 0(0) |  |
| No | 65(98.5) | 43(78.2) |  | 14(93.3) | 15(100.0) |  |
| Tumor Number |  |  | 0.516 |  |  | - |
| Single | 64(97.0) | 52(92.7) |  | 15(100.0) | 15(100.0) |  |
| Multiple | 2(3.0) | 4(7.3) |  | 0(0) | 0(0) |  |
| T Stage |  |  | 0.150 |  |  | 0.699 |
| T1 | 36(54.5) | 25(45.5) |  | 11(73.3) | 9(60.0) |  |
| T2 | 26(39.4) | 30(54.5) |  | 4(26.7) | 6(40.0) |  |
| T3 | 1(1.5) | 0(0) |  | 0(0) | 0(0) |  |
| T4 | 3(4.5) | 0(0) |  | 0(0) | 0(0) |  |
| Grade^‡^ |  |  | 0.022 |  |  | 0.133 |
| G1-G2 | 21(31.8) | 27(49.1) |  | 4(26.7) | 9(60.0) |  |
| G3-G4 | 27(40.9) | 10(18.2) |  | 5(33.3) | 4(26.7) |  |
| Unknown | 18(27.3) | 18(32.7) |  | 6(40.0) | 2(13.3) |  |
| Metavir Stage |  |  | 0.002 |  |  | 0.680 |
| F0-F3 | 13(19.7) | 0(0) |  | 0(0) | 0(0) |  |
| F4 | 9(13.6) | 13(23.6) |  | 3(20.0) | 5(33.3) |  |
| Unknown | 44(66.7) | 42(76.4) |  | 12(80.0) | 10(66.7) |  |

Hx=Hepatectomy; LT=Liver transplantation; PSM=Propensity score matching; CHC=Combined hepatocellular-cholangiocarcinoma; AFP=Alpha fetoprotein.

^†^U.S. Census Bureau, Real Median Household Income in the United States [MEHOINUSA672N], retrieved from FRED, Federal Reserve Bank of St. Louis; https://fred.stlouisfed.org/series/MEHOINUSA672N, June 26, 2021

^‡^G1=Well differentiated; G2=Moderately differentiated; G3-4=Poorly differentiated/Undifferentiated

**Table S5**. Comparison between Hx and LT before and after PSM in CHC patients beyond the Milan Criteria

| Factors | Before PSM | | | After PSM | | |
| --- | --- | --- | --- | --- | --- | --- |
|  | Hx  (n=102) | LT  (n=9) | P | Hx  (n=9) | LT  (n=9) | P |
| Year of Diagnosis |  |  | 0.547 |  |  | 1.000 |
| 2004-2011 | 40(39.2) | 5(55.6) |  | 4(44.4) | 5(55.6) |  |
| 2012-2018 | 62(60.8) | 4(44.4) |  | 5(55.6) | 4(44.4) |  |
| Age |  |  | 0.034 |  |  | 1.000 |
| ≤60 | 47(46.1) | 8(88.9) |  | 8(88.9) | 8(88.9) |  |
| >60 | 55(53.9) | 1(11.1) |  | 1(11.1) | 1(11.1) |  |
| Sex |  |  | 0.630 |  |  | 1.000 |
| Female | 37(36.3) | 2(22.2) |  | 2(22.2) | 2(22.2) |  |
| Male | 65(63.7) | 7(77.8) |  | 7(77.8) | 7(77.8) |  |
| Race |  |  | 0.724 |  |  | 0.260 |
| White | 74(72.5) | 8(88.9) |  | 5(55.6) | 8(88.9) |  |
| Asia-Pacific | 20(19.6) | 1(11.1) |  | 3(33.3) | 1(11.1) |  |
| Black | 7(6.9) | 0(0) |  | 1(11.1) | 0(0) |  |
| Other | 1(1.0) | 0(0) |  | 0(0) | 0(0) |  |
| Residence |  |  | 1.000 |  |  | 1.000 |
| Urban | 90(88.2) | 8(88.9) |  | 9(100.0) | 8(88.9) |  |
| Rural | 12(11.8) | 1(11.1) |  | 0(0) | 1(11.1) |  |
| Income^†^ |  |  | 0.507 |  |  | 0.617 |
| Below the median | 63(61.8) | 4(44.4) |  | 2(22.2) | 4(44.4) |  |
| Above the median | 39(38.2) | 5(55.6) |  | 7(77.8) | 5(55.6) |  |
| AFP |  |  | 0.175 |  |  | 0.801 |
| Negative | 25(24.5) | 1(11.1) |  | 2(22.2) | 1(11.1) |  |
| Positive | 50(49.0) | 3(33.3) |  | 3(33.3) | 3(33.3) |  |
| Borderline/Unknown | 27(26.5) | 5(55.6) |  | 4(44.4) | 5(55.6) |  |
| First Malignant |  |  | 1.000 |  |  | 1.000 |
| Yes | 89(87.3) | 8(88.9) |  | 8(88.9) | 8(88.9) |  |
| No | 13(12.7) | 1(11.1) |  | 1(11.1) | 1(11.1) |  |
| Neoadjuvant Therapy |  |  | 0.066 |  |  | 0.453 |
| Yes | 3(2.9) | 2(22.2) |  | 0(0) | 2(22.2) |  |
| No | 99(97.1) | 7(77.8) |  | 9(100.0) | 7(77.8) |  |
| Tumor Number |  |  | 0.555 |  |  | 1.000 |
| Single | 92(90.2) | 7(77.8) |  | 7(77.8) | 7(77.8) |  |
| Multiple | 10(9.8) | 2(22.2) |  | 2(22.2) | 2(22.2) |  |
| Tumor Size |  |  | 0.029 |  |  | 1.000 |
| ≤5cm | 12(11.8) | 4(44.4) |  | 4(44.4) | 4(44.4) |  |
| >5cm | 90(88.2) | 5(55.6) |  | 5(55.6) | 5(55.6) |  |
| T Stage |  |  | 0.470 |  |  | 0.815 |
| T1 | 28(27.5) | 1(11.1) |  | 1(11.1) | 1(11.1) |  |
| T2 | 47(46.1) | 7(77.8) |  | 6(66.7) | 7(77.8) |  |
| T3 | 5(4.9) | 0(0) |  | 0(0) | 0(0) |  |
| T4 | 4(3.9) | 0(0) |  | 0(0) | 0(0) |  |
| TX | 18(17.6) | 1(11.1) |  | 2(22.2) | 1(11.1) |  |
| N Stage |  |  | 0.829 |  |  | 0.445 |
| N0 | 73(71.6) | 6(66.7) |  | 8(88.9) | 6(66.7) |  |
| N1 | 15(14.7) | 2(22.2) |  | 1(11.1) | 2(22.2) |  |
| NX | 14(13.7) | 1(11.1) |  | 0(0) | 1(11.1) |  |
| M Stage |  |  | 0.859 |  |  | 1.000 |
| M0 | 94(92.2) | 8(88.9) |  |  | 8(88.9) |  |
| M1 | 7(6.9) | 1(11.1) |  | 1(11.1) | 1(11.1) |  |
| MX | 1(1.0) | 0(0) |  | 0(0) | 0(0) |  |
| Grade^‡^ |  |  | 0.379 |  |  | 0.347 |
| G1-G2 | 34(33.3) | 5(55.6) |  | 2(22.2) | 5(55.6) |  |
| G3-G4 | 57(55.9) | 3(33.3) |  | 5(55.6) | 3(33.3) |  |
| Unknown | 11(10.8) | 1(11.1) |  | 2(22.2) | 1(11.1) |  |
| Metavir Stage |  |  | 0.415 |  |  | 0.117 |
| F0-F3 | 16(15.7) | 0(0) |  | 3(33.3) | 0(0) |  |
| F4 | 7(6.9) | 1(11.1) |  | 0(0) | 1(11.1) |  |
| Unknown | 79(77.5) | 8(88.9) |  | 6(66.7) | 8(88.9) |  |

Hx=Hepatectomy; LT=Liver transplantation; PSM=Propensity score matching; CHC=Combined hepatocellular-cholangiocarcinoma; AFP=Alpha fetoprotein.

^†^U.S. Census Bureau, Real Median Household Income in the United States [MEHOINUSA672N], retrieved from FRED, Federal Reserve Bank of St. Louis; https://fred.stlouisfed.org/series/MEHOINUSA672N, June 26, 2021

^‡^G1=Well differentiated; G2=Moderately differentiated; G3-4=Poorly differentiated/Undifferentiated

**Table S6**. Comparison between CHC patients with LT within and beyond the Milan Criteria

| Factors | Within the Milan Criteria  (n=55) | Beyond the Milan Criteria  (N=9) | P |
| --- | --- | --- | --- |
| Year of Diagnosis |  |  | 0.926 |
| 2004-2011 | 35(63.6) | 5(55.6) |  |
| 2012-2018 | 20(36.4) | 4(44.4) |  |
| Age |  |  | 0.410 |
| ≤60 | 38(69.1) | 8(88.9) |  |
| >60 | 17(30.9) | 1(11.1) |  |
| Sex |  |  | 1.000 |
| Female | 13(23.6) | 2(22.2) |  |
| Male | 42(76.4) | 7(77.8) |  |
| Race |  |  | 0.610 |
| White | 46(83.6) | 8(88.9) |  |
| Asia-Pacific | 4(7.3) | 1(11.1) |  |
| Black | 5(9.1) | 0(0) |  |
| Residence |  |  | 1.000 |
| Urban | 49(89.1) | 8(88.9) |  |
| Rural | 6(10.9) | 1(11.1) |  |
| Income^†^ |  |  | 0.839 |
| Below the median | 30(54.5) | 4(44.4) |  |
| Above the median | 25(45.5) | 5(55.6) |  |
| AFP |  |  | 0.122 |
| Negative | 18(32.7) | 1(11.1) |  |
| Positive | 24(43.6) | 3(33.3) |  |
| Borderline/Unknown | 13(23.6) | 5(55.6) |  |
| First Malignant |  |  | 0.605 |
| Yes | 41(74.5) | 8(88.9) |  |
| No | 14(25.5) | 1(11.1) |  |
| Primary Tumor |  |  | 0.577 |
| Yes | 48(87.3) | 9(100.0) |  |
| No | 7(12.7) | 0(0) |  |
| Neoadjuvant Therapy |  |  | 1.000 |
| Yes | 12(21.8) | 2(22.2) |  |
| No | 43(78.2) | 7(77.8) |  |
| Tumor Number |  |  | 0.418 |
| Single | 52(92.7) | 7(77.8) |  |
| Multiple | 4(7.3) | 2(22.2) |  |
| Tumor Size |  |  | <0.001 |
| ≤5cm | 55(100.0) | 4(44.4) |  |
| >5cm | 0(0) | 5(55.6) |  |
| T Stage |  |  | 0.011 |
| T1 | 25(45.5) | 1(11.1) |  |
| T2 | 30(54.5) | 7(77.8) |  |
| TX | 0(0) | 1(11.1) |  |
| N Stage |  |  | <0.001 |
| N0 | 55(100.0) | 6(66.7) |  |
| N1 | 0(0) | 2(22.2) |  |
| NX | 0(0) | 1(11.1) |  |
| M Stage |  |  | 0.297 |
| M0 | 55(100.0) | 8(88.9) |  |
| M1 | 0(0) | 1(11.1) |  |
| Grade^‡^ |  |  | 0.340 |
| G1-G2 | 27(49.1) | 5(55.6) |  |
| G3-G4 | 10(18.2) | 3(33.3) |  |
| Unknown | 18(32.7) | 1(11.1) |  |
| Mtavir Stage |  |  | 0.683 |
| F4 | 13(23.6) | 1(11.1) |  |
| Unknown | 42(76.4) | 8(88.9) |  |

Hx=Hepatectomy; LT=Liver transplantation; PSM=Propensity score matching; CHC=Combined hepatocellular-cholangiocarcinoma; AFP=Alpha fetoprotein.

^†^U.S. Census Bureau, Real Median Household Income in the United States [MEHOINUSA672N], retrieved from FRED, Federal Reserve Bank of St. Louis; https://fred.stlouisfed.org/series/MEHOINUSA672N, June 26, 2021

^‡^G1=Well differentiated; G2=Moderately differentiated; G3-4=Poorly differentiated/Undifferentiated
